# Comparative analysis of ear-hole closure identifies epimorphic regeneration as a discrete trait in mammals

**DOI:** 10.1038/ncomms11164

**Published:** 2016-04-25

**Authors:** Thomas R. Gawriluk, Jennifer Simkin, Katherine L. Thompson, Shishir K. Biswas, Zak Clare-Salzler, John M. Kimani, Stephen G. Kiama, Jeramiah J. Smith, Vanessa O. Ezenwa, Ashley W. Seifert

**Affiliations:** 1 Department of Biology, University of Kentucky, Lexington, Kentucky 40506, USA; 2 Department of Statistics, University of Kentucky, Lexington, Kentucky 40506, USA; 3 Department of Veterinary Anatomy and Physiology, University of Nairobi, PO Box 30197-00100, Nairobi, Kenya; 4 Odum School of Ecology, University of Georgia, Athens, Georgia 30602, USA; 5 Department of Infectious Disease, University of Georgia, Athens, Georgia 30602, USA

## Abstract

Why mammals have poor regenerative ability has remained a long-standing question in biology. In regenerating vertebrates, injury can induce a process known as epimorphic regeneration to replace damaged structures. Using a 4-mm ear punch assay across multiple mammalian species, here we show that several *Acomys spp*. (spiny mice) and *Oryctolagus cuniculus* completely regenerate tissue, whereas other rodents including MRL/MpJ ‘healer' mice heal similar injuries by scarring. We demonstrate ear-hole closure is independent of ear size, and closure rate can be modelled with a cubic function. Cellular and genetic analyses reveal that injury induces blastema formation in *Acomys cahirinus*. Despite cell cycle re-entry in *Mus musculus* and *A. cahirinus*, efficient cell cycle progression and proliferation only occurs in spiny mice. Together, our data unite blastema-mediated regeneration in spiny mice with regeneration in other vertebrates such as salamanders, newts and zebrafish, where all healthy adults regenerate in response to injury.

## Abstract (web summary)

The extent to which mammals and other vertebrates share similar mechanisms of tissue regeneration is unclear. Here, the authors use an ear punch assay in spiny mice, which regenerate fully, to show blastema formation and mesenchymal cell proliferation as cell cycle regulators p21 and p27 remain cytoplasmic.

## Graphical abstract

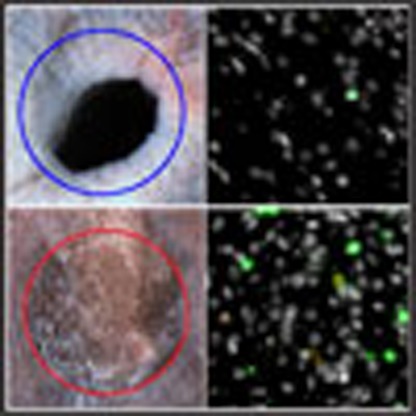

We previously reported that two species of wild African spiny mice, *Acomys kempi* and *A. percivali*, regenerate full-thickness skin injuries^1^. In addition, these species completely closed circular through-and-through wounds in the ear pinna and regenerated the excised tissue. An outbred, laboratory mouse strain (*Mus musculus*, Swiss Webster), on the other hand, failed to close identical-sized ear holes and healed these wounds by scarring^1^. These results suggested that ear-hole closure could broadly predict regenerative ability among mammals, although the extent to which other taxa, including additional *Acomys* species, might possess the ability to regenerate injured tissue remains unknown. Another unanswered question is whether *Acomys* form a blastema and undergo epimorphic regeneration similar to regeneration observed during salamander, newt and zebrafish appendage regeneration. During blastema-mediated epimorphic regeneration, locally recruited progenitor cells proliferate to form a heterogeneous mass of cells that subsequently undergo differentiation to regenerate the missing structures. This process is different from epimorphic regeneration that occurs through the direct recruitment and proliferation of differentiated cells as seen during skin regeneration, or morphallaxis, where de-differentiation, rearrangement and differentiation of local cells occurs before proliferation.

In the current study we set out to (1) test the ability of several mammalian species to close a 4-mm ear hole and regenerate the excised tissue, (2) test the hypothesis that ear-hole closure can distinguish species capable of regenerating ear tissue from those that heal by scarring, (3) quantify intra- and interspecific differences in ear-hole closure rate, (4) test the hypothesis that *A. cahirinus* form a mammalian blastema to regenerate ear tissue and (5) assess the extent to which ear pinna cells can re-enter the cell cycle, undergo cell cycle progression and proliferate in response to injury in *A. cahirinus* and *M. musculus*.

## Results

### 4-mm ear punch identifies regenerative ability in mammals

**Figure 1:**
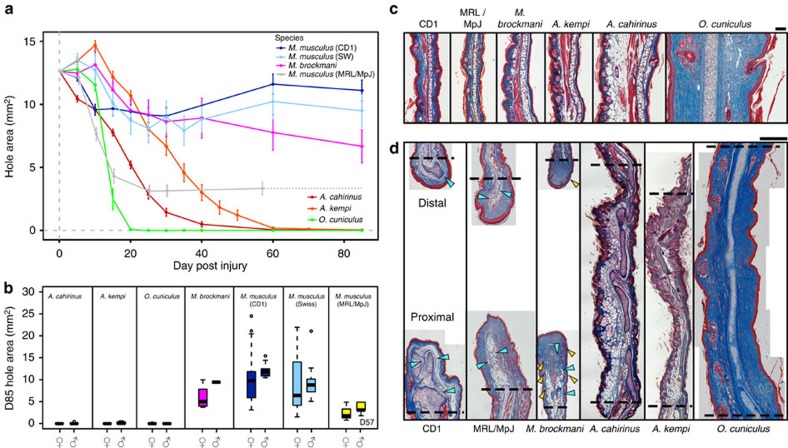
Complete ear-hole closure is coupled with tissue regeneration. (**a**) Comparison of 4-mm ear-hole punch assay for indicated species indicating ear-hole area over time (data are for one ear per individual and include males and females). For MRL/MpJ dotted line indicates extrapolated data from a last measurement on D57. Data represent mean±s.e.m. (**b**) Ear-hole area at D85 (D57 for MRL/MpJ) for indicated sex and species. Species cluster into those that do not close ear holes (*M. musculus*—CD1, Swiss Webster and MRL/MpJ, and *M. brockmani*) and those that completely close ear holes (*A. kempi*, *A. cahirinus* and *O. cuniculus*). Data shown as box and whiskers where central line represents median, whiskers represent the range (excluding outliers, which are represented by circles) and the box represents the middle 50% of the data. See Supplementary Table 1 for experimental animal sample sizes (**a**,**b**). (**c**) Representative images of uninjured (D0) ear pinna. The dorsal compartment (left) and ventral compartment (right) are separated by elastic cartilage. Tissue is stained with Masson's trichrome: muscle (red); cytoplasm (pink); collagen (blue); and nuclei (black). Scale bar, 100 μM. (**d**) Representative images of ear pinna tissue at D85 showing open ear holes and scar tissue formation in *M. musculus* (CD1 and MRL/MpJ) and *M. brockmani* and closed holes and tissue regeneration in *A. kempi*, *A. cahirinus* and *O. cuniculus*. New cartilage in non-regenerating species is disorganized and mis-patterned (blue arrows), whereas new cartilage in regenerating species is normally patterned across the entire ear. Hair follicles and sebaceous glands are present in regenerating species and *M. brockmani* (yellow arrows). Amputation planes (dotted line) are indicated. Scale bar, 500 μM. The represented histological sections from a minimum of *n*=3 biological replicates show cross-sections through the middle of the wound along the proximal–distal axis, including uninjured tissue and the amputation planes.

First, we used a 4-mm ear punch assay to assess whether species could be separated by their ability to close an ear hole (Fig. 1a,b). We studied a total of five species (or seven groups including three strains of *M. musculus*): *A. kempi* (one of the species in our original report) that inhabits rocky areas across the dry savannas of Kenya, Somalia, Ethiopia and Tanzania; *A. cahirinus*, indigenous to rocky habitats across Northern Africa and the Middle East; *Myomyscus brockmani*, a murid rodent that is sympatric with *A. kempi*; two outbred laboratory strains of *M. musculus*, CD1 (obtained in USA) and Swiss Webster (obtained in Kenya); one inbred strain of *M. musculus*, MRL/MpJ (Murphy's Large Roth); and a rabbit, *Oryctolagus cuniculus* (New Zealand White; Supplementary Table 1). Extending our original findings, we found *A. kempi* completely closed 4-mm ear holes (that is, tissue filled in the entire hole area) through the pinna. By D85 of the experiment, all female *A. kempi* (12/12) and two males (2/4) completely closed ear holes (Fig. 1a,b and Supplementary Fig. 1a,b), while ear holes in the remaining two males were nearly closed (unclosed ear-hole areas at D85=0.6 and 0.2 mm^2^, respectively). Likewise, we found that for congeneric *A. cahirinus*, every female (26/26) and all but one male (13/14) completely closed ear holes by D85 (unclosed ear-hole area at D85=0.5 mm^2^; Fig. 1a,b), with the remaining male closing its ear hole by D100. In addition to these spiny mice, male (10/10) and female (8/9) *O. cuniculus* completely closed 4-mm ear holes by D85, with the outstanding female almost entirely closed by that time (unclosed ear-hole area at D85=0.2 mm^2^; Fig. 1a,b and Supplementary Fig. 1 a,b). By comparison, none of the outbred strains of *M. musculus* (0/57) or *M. brockmani* (0/5) completely closed 4-mm ear holes (Fig. 1a,b). Similarly, none of the Murphy's Roth Large (MRL/MpJ) ‘healer mice' (0/10) completely closed 4-mm ear holes (Fig. 1a,b). Thus, of the species tested, our data demonstrate that only *Acomys spp.* and *O. cuniculus* could completely close 4-mm ear holes.

**Figure 2:**
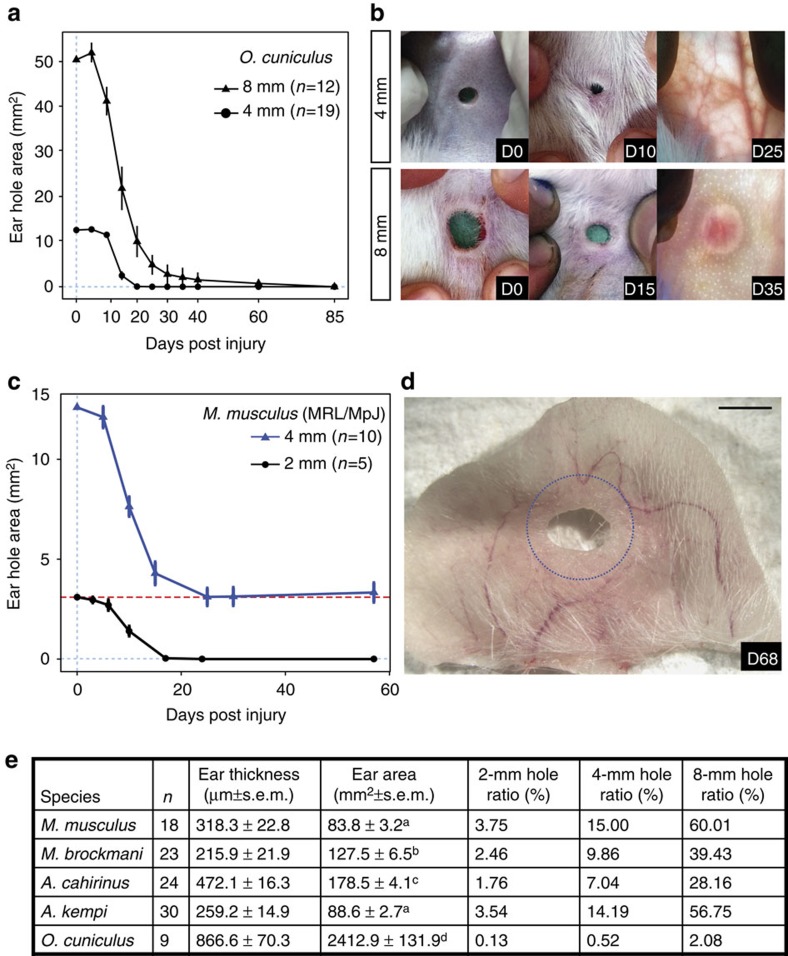
Ear pinna regeneration is not dependent on hole size. (**a**,**c**) Ear-hole area over time after an 8- (triangles) and 4-mm (circles) ear-hole punch assay in New Zealand white rabbits (**a**) and after a 4- (blue) and 2-mm (black) ear-hole punch assay in MRL/MpJ mice (**c**). Dotted lines were placed at observed asymptotes for closure, clearly showing that 4- and 8-mm holes close completely in rabbits (**a**), and only 2- but not 4-mm holes close completely in MRL/MpJ (**c**). Data represent mean and s.e.m. with lines connecting the means, and *n* is the total number of individuals observed (**a**,**c**). (**b**,**d**) Representative images of rabbit and MRL/MpJ ear holes in response to injury. Scale bar, 2 mm, and blue dotted line is original 4-mm hole (**d**). (**e**) Summary data indicating that ear size is not associated with ability for ear closure. Individual ears were traced onto paper and digitalized traces were used to determine ear area using ImageJ. The cross-sectional distance from the outer edges of the stratum corneum was measured from histology at D0 to determine ear thickness. For ear area, separate superscripts (a versus b, a versus c and b versus c) represent a *P* value <0.05 for Tukey *post hoc* comparison.

Although we confirmed previous reports of ear-hole closure in rabbits^2^,^3^, our failure to do so in inbred MRL/MpJ mice suggested that wound size might affect closure ability. To test whether ear-hole size impacted regenerating species, we created a larger, 8-mm hole in *O. cuniculus* and found they completely closed these holes by D85 (Fig. 2a,b). We also asked if *A. cahirinus* could close 8-mm ear holes. Although 8-mm ear holes often tore (preventing analysis), those that did not tear completely closed before D150 (Supplementary Fig. 2). While MRL/MpJ completely closed 2-mm ear punches within 21 days similar to previous reports^4^,^5^, closure of 4-mm ear punches in MRL/MpJ animals plateaued to approximately the area of a 2-mm punch in the same amount of time (Fig. 2c,d). Furthermore, 5.3% (3/57) of the outbred *M. musculus* closed 4-mm ear holes to an area less than or equal to the area of a 2-mm ear punch (Supplementary Fig. 1a). To evaluate whether ear size could explain differences in closure ability, we calculated the ratio of ear-hole size as a fraction of total ear area (ear-hole ratio; Fig. 2e). While ear size was variable across species, the data show that this ratio does not predict ear-hole closure ability. For instance, ear-hole ratios for a 2-, 4- and 8-mm hole were the same for *A. kempi* and outbred *M. musculus* even though the former completely closed their wounds while the latter never did. On the other hand, *M. brockmani* had a smaller ear-hole ratio compared with *A. kempi* for similar holes and thus, if this ratio predicted ear-hole closure ability, then *M. brockmani* should have completely close ear holes, which they did not.

These data support that a sufficiently large enough ear punch is necessary to reliably and reproducibly separate mammalian species into those that can and those that cannot close ear punches. Importantly, nearly all African spiny mice and rabbits closed 4-mm ear holes by D85 (that is, there was no variation among individuals with respect to closure ability; Supplementary Fig. 1a,b). In stark contrast, closure among *M. musculus* strains and *M. brockmani* was variable, but none of these animals closed 4-mm ear holes (Supplementary Fig. 1a,b). Together, these data suggested that the ability to completely close 4-mm ear holes is a unique trait because variation across these two groups was discontinuous.

### Complete hole closure is coupled with tissue regeneration

Our observations of pigment and hair follicles in new tissue of spiny mice and rabbits suggested that complete ear-hole closure occurred through the regeneration of new tissue, whereas partial ear-hole closure in other species occurred through the fast accumulation of scar tissue. To test this hypothesis, we compared the architecture of uninjured D0 and injured D85 (D96 in MRL/MpJ) tissue from each of the above species/strains. Uninjured ear tissue across rodents had a similar cellular organization (Fig. 1c). Across species, the ear pinna was divided dorsoventrally by a piece of elastic cartilage with overlying (dorsal) tissues being thicker and containing a larger number of epidermally derived appendages relative to ventral tissues (that is, hair follicles and sebaceous glands; Fig. 1c). In *Acomys spp.*, there appeared to be a greater number of ventral sebaceous glands compared with the other rodents (Fig. 1c). The dorsal compartment of the ear also contained a layer of muscle and adipose tissue (Fig. 1c). Compared with the rodents, the rabbit ear pinna had a uniform distribution of appendages in the dorsal and ventral compartments (Fig. 1c). There was no muscle in the excised region, and the rabbit dermis was comparatively thicker with more densely layered collagen fibres. Thus, the ear tissue was morphologically similar before injury across species.

Analysis of D85 tissue supported the hypothesis that *Acomys spp.* and *O. cuniculus* regenerated tissue after ear injury and that *M. musculus* strains and *M. brockmani* healed by scarring (Fig. 1d). We observed several marked differences between regenerating species and non-regenerating species. We first assessed the alignment and cellular arrangement of new cartilage that was located distal to the original, uncut cartilage. While there was evidence of new cartilage in non-regenerating species, it appeared as disorganized nodules compared with the new, centrally aligned cartilage in regenerating species (Fig. 1d). In *M. musculus* and *M. brockmani*, new cartilage developed into nodules that were often round in cross-section, uncharacteristically thick, and in some cases, these nodules branched or developed into disorganized masses (Fig. 1d). In contrast, both *Acomys spp.* and *O. cuniculus* developed new cartilage centrally within the ear pinna, in line and of similar thickness with the original, uninjured cartilage (Fig. 1d). By D85, this regenerated cartilage had reconstituted a majority of the excised cartilaginous sheet and chondrocytes appeared in the process of completely differentiating to form elastic cartilage (Fig. 1d).

**Figure 3:**
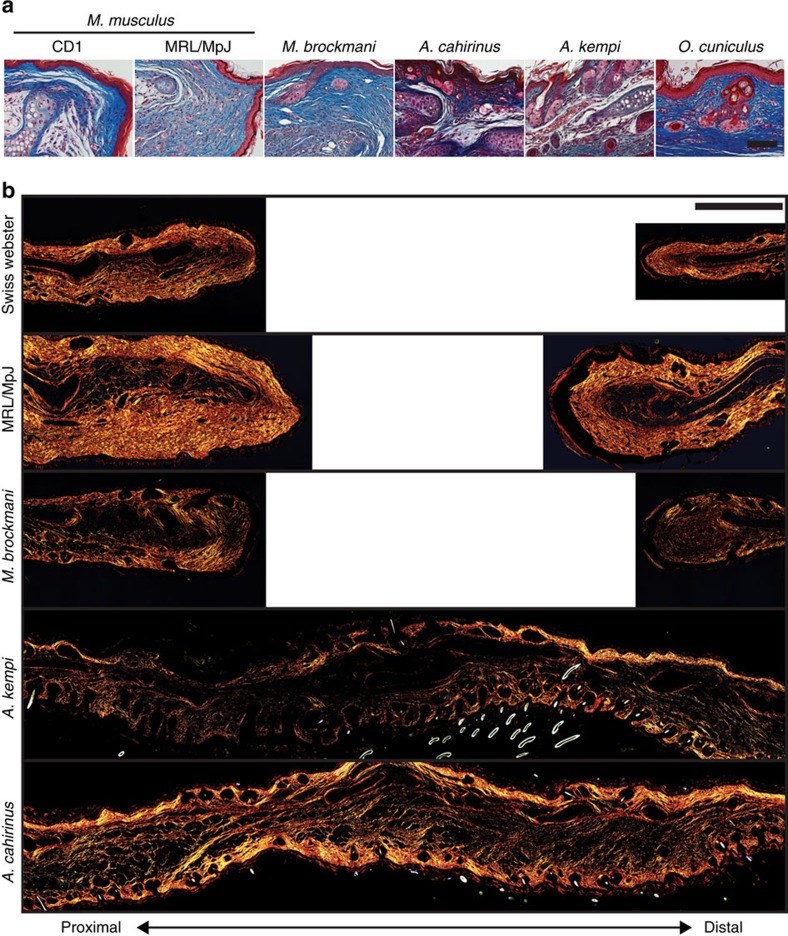
Regenerating species restore dermal architecture whereas non-regenerating species form scar tissue in the dermis. (**a**) Representative images showing the dermis at D85 (D96 for MRL/MpJ) stained with Masson's trichrome. Regenerating species (for example, *A. cahirinus*, *A. kempi* and *O. cuniculus*) regenerate full-thickness skin, including bilayer dermis and epidermis with hair follicles and sebaceous glands. Non-regenerators (for example, *M. musculus* strains—CD1 and MRL/MpJ, and *M. brockmani*) form scar tissue beneath the epidermis evident as dense, parallel bands of collagen. In *Mus* strains, no new hair follicles or sebaceous glands are evident distal to the wound. In contrast, new hair follicles and sebaceous glands are present in *M. brockmani*. (**b**) Representative images of D85 tissue stained with picrosirius red viewed under polarized light to visualize thick (red) and thin (green) collagen fibres. In non-regenerating species the thick, parallel fibres with very little observed thin fibres were indicative of a scar. However, in regenerating species, collagen architecture is more representative of uninjured collagen pattern of a thick and thin network of uninjured tissue. All images are representative of at least *n*=3 biological replicates per species. Scale bars, 50 (**a**) and 500 μM (**b**).

To assess fibrotic scarring, we analysed the dermis at D85 and found no evidence for scarring in *Acomys spp.* and *O. cuniculus* (Figs 1d and Fig. 3a,b). Spiny mice and rabbits exhibited a uniform extracellular matrix that appeared similar to unwounded dermis (Figs 1d and 3a). In fact, the dermis re-developed its original architecture complete with hair follicles and sebaceous glands in these regenerating species (Figs 1d and 3a). In contrast, we found evidence of scarring in all of the non-regenerators, with collagen deposited in whorl-like patterns and as dense parallel bands in the repaired tissue (Figs 1d and 3a). Picrosirius red staining at D85 showed that collagen was present in densely packed, parallel bundles in the dermis of non-regenerators, whereas regenerators showed the characteristic unwounded pattern of collagen (Fig. 3b). While we rarely observed hair follicles or sebaceous glands beyond the proximal amputation plane in non-regenerating species, *M. brockmani* regenerated sebaceous glands and pairs of hair follicles beyond the proximal amputation plane (Fig. 1d). The data reinforce that broader sampling across wild mammal species has the potential to uncover additional examples of tissue regeneration. Together, our results using a 4-mm wound in the pinna establish that complete ear-hole closure is coupled with tissue regeneration, while the failure to close an ear hole is associated with fibrosis and scarring.

### Quantitative modelling reveals variation in regeneration rate

Given obvious qualitative differences in regeneration ability between regenerating and non-regenerating species, we next set out to examine interspecific differences in closure rate. To accomplish this, we performed a quantitative analysis of closure using *A. cahirinus*, *A. kempi*, *M. brockmani* and the two *M. musculus* strains, CD1 and Swiss Webster. We tested the hypotheses that the kinetics of closure differs by regeneration phenotype (that is, regenerator versus non-regenerator), and that within regenerators there are differences in regeneration rate associated with traits such as sex and reproductive status. We modelled closure, measured as ear-hole area from D5 through D30, using a repeated measures analysis of variance (ANOVA) with species, sex and day^3^ as main effects, and day^3^ × species, day^3^ × sex, species × sex and day^3^ × species × sex as interaction effects (see Methods).

**Figure 4:**
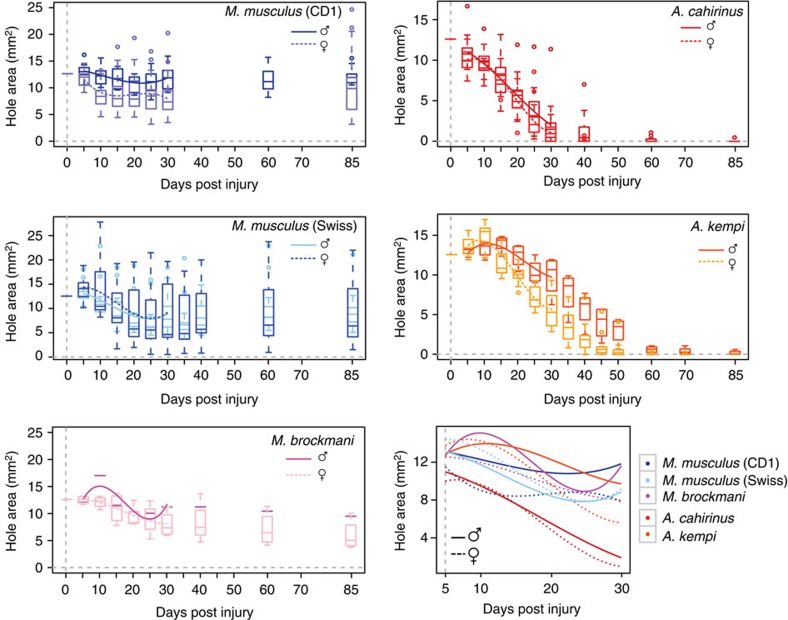
A cubic function describes ear closure across species. Ear-hole area modelled over time following a 4-mm ear-hole punch for each species (box and whiskers). The estimated cubic function is overlaid from D5–D30, and the data are graphed separately for each sex (male=solid line and female=dashed line). For comparison, all cubic functions are overlaid in bottom right panel.

Our model of ear-hole closure revealed a significant interaction between the cubic effect of time (day), sex and species (repeated measures ANOVA, *F*=6.58, Huynh–Feldt–Lecoutre (H–F–L) *P*<0.0001), with ear-hole area tending to increase initially and then decrease over time for all species (Fig. 4). This significant three-way interaction between day^3^, species and sex indicated that species varied in their closure rates, and that closure rate differed by sex in at least some species Table 1. As could be predicted based on ability, when we tested for differences in closure rate in our model across all species pairs, regenerating and non-regenerating species could be separated (Table 2). Among the regenerators, *A. cahirinus* regenerated faster than *A. kempi* (Supplementary Fig. 3a). Among the non-regenerators, closure rates were significantly different between CD1 and Swiss Webster mice for both sexes (Table 2). By contrast, *M. brockmani* did not differ from either *M. musculus* strain (Table 2). The difference we observed between pairs of *Acomys spp.* and *M. musculus* might reflect differences in the environmental conditions under which the punch assays were performed. We performed our experiments on *A. cahirinus* and CD1 in the USA (Lexington, Kentucky) and on *A. kempi* and Swiss Webster in Kenya (Nairobi) and thus, differences in abiotic conditions (for example, temperature, humidity, altitude and so on) could have affected ear-hole closure rate during both regeneration and scarring. However, it is equally possible that variation in unmeasured biotic factors such as food, pathogen infections, genetic variation and so on might have contributed to the observed difference.

We next asked if sex could explain within-species variation among regenerators. Interestingly, there was no difference in closure rate between male and female *A. cahirinus*, while within *A. kempi*, females regenerated faster than males (repeated measures ANOVA, *F*=8.73; H–F–L *P*=0.0003; Supplementary Table 2). Since the *A. kempi* in our experiment had a small volume of blood removed (∼100 μl) at D0, D1 and D15, we tested for sex-specific effects of blood sampling on ear-hole closure by utilizing our colony of *A. cahirinus* and comparing blood-sampled individuals with controls (BC Cahirinus group in Supplementary Table 1). Using our ear closure model, we detected a significant interaction between day, sex and blood collection (repeated measures ANOVA, *F*=3.90, H–F–L *P*=0.0066). Analysing males and females with and without blood collected showed significant differences in ear-hole closure across time in both sexes (repeated measures ANOVA, males: *F*=12.47, H–F–L *P*<0.0001; females: *F*=34.85, H–F–L *P*<0.0001). Blood collection caused a delay in the onset of the regeneration response in males and females, compared with controls (Supplementary Fig. 3b). We also found that blood collection led to faster closure rate in females compared with control animals and blood-collected males (repeated measures ANOVA, *F*=4.72; H–F–L *P*=0.0058). In contrast, there was no significant difference in ear-hole closure by sex when blood was not collected (repeated measures ANOVA, *F*=2.16; H–F–L *P*=0.0824). Thus, blood collection seems to have revealed a sex bias in regeneration rate between male and female *A. cahirinus* that mirrors the sex bias we observed in *A. kempi* (Supplementary Fig. 3c). In addition to sex, we also tested for effects of female reproductive status (pregnancy and lactation) on ear-hole closure rate within *A. cahirinus* and found that lactation produced a small increase in ear-hole regeneration rate from D5–30 compared with non-lactating and non-pregnant controls (Supplementary Fig. 3c and Supplementary Table 3). Interestingly, lactation did not affect time to closure (Supplementary Fig. 3d), suggesting that the slight increase in closure rate seen with lactation is not associated with a biologically significant effect. Together, our results suggest that when female *A. cahirinus* experience physiological challenge such as milk production or small volume blood loss, regeneration rate increases.

### Transcriptomic analysis of regeneration and scarring

**Figure 5:**
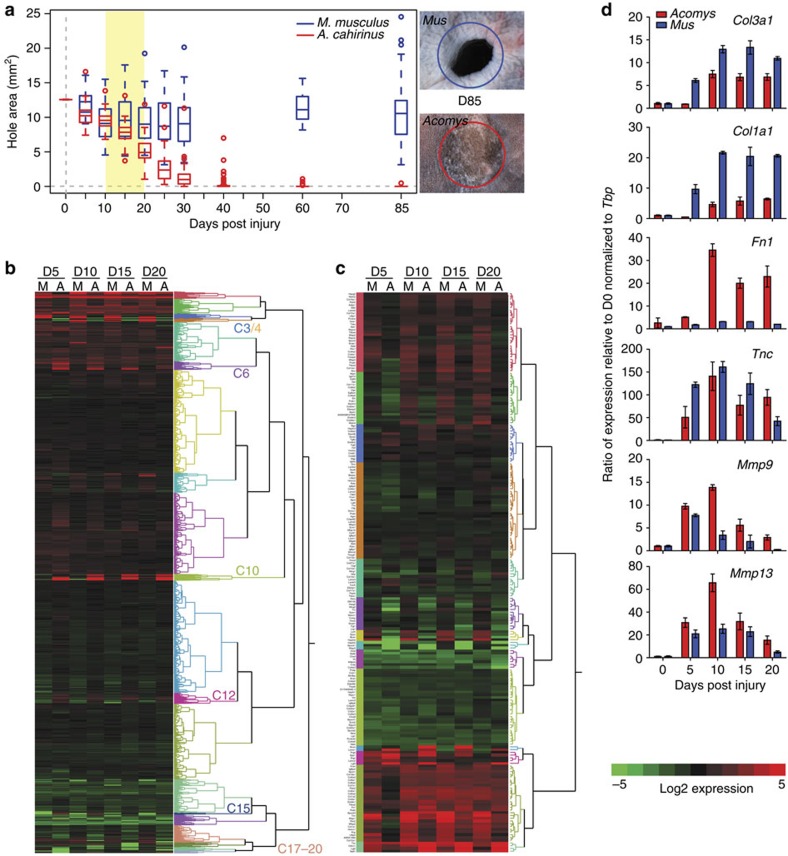
Analysis of global transcription reveals a regeneration-specific transcriptional response associated with ECM-related genes. (**a**) Ear-hole area following 4-mm ear-hole punch assay in *M. musculus* (blue) and *A. cahirinus* (red). Data represent box and whisker plots from male and female individuals re-plotted from Fig. 1a. Adjacent images of representative ears at D85 for *M. musculus* (top) and *A. cahirinus* (bottom). Blue and red circles indicate initial wound. Regenerated hair follicles are present in *A. cahirinus*. (**b**) Heatmap showing expression relative to D0 for global transcription from *M. musculus* (M) and *A. cahirinus* (A) at D5, D10, D15 and D20. Tree to the right of heatmap represents the ontogeny of gene expression and each colour is used to separate the genes into 20 unique clusters via hierarchical clustering based on expression pattern (C3/C4=up in M, no change in A; C6/C10=no change in M, up in A; C12=down in M, up in A; C15=down in M, up in A; C17/C18/C19/C20=no change in M, down in A). (**c**) Heatmap for 204 out of 277 genes from the core murine matrisome (glycoproteins, collagens and proteoglycans). Data represent log_2_(mean expression) calculated from EBseq averaged over *n*=3 biological replicates at each time point per species (**b**,**c**). (**d**) Quantitative PCR results for select genes from matrisome. Data represent mean and s.e.m. for *n*=3 biological replicates at each time point per species.

To identify the cellular and molecular mechanisms underlying a mammalian regenerative response, we performed a controlled comparison between regenerating *A. cahirinus* and scarring *M. musculus* (Fig. 5a). Following the wound after injury in *A. cahirinus* and *M. musculus,* we observed a similar haemostatic response. Red blood cells moved into the interstitial space and this resulted in a blood clot at the injury site (Supplementary Fig. 4a,b). Haemostasis was followed by an inflammatory response that included the infiltration of neutrophils and monocytes as evidenced by F4/80 immunostaining (Supplementary Fig. 4c,d). Local tissue underwent histolysis, the collagen-rich extracellular matrix (ECM) fragmented and the epidermis migrated over the edge of the cartilage plate as re-epithelialization commenced (Supplementary Fig. 4a,b). Although re-epithelialization in *Acomys* occurred more slowly compared with *Mus*, the early response to injury was similar in both species.

To gain mechanistic insight into the molecular control of regeneration, we performed next-generation sequencing on RNA from healing tissue at D0, D5, D10, D15 and D20 to identify global differences in gene expression between regenerating and scar-forming tissues in *A. cahirinus* and *M. musculus*, respectively (Fig. 5b). Using our computational pipeline (see Methods), we detected 14,735 orthologous genes expressed in both species, which were grouped into 20 clusters based on their expression patterns (Fig. 5b and Supplementary Data 1). An unbiased analysis showed that a simplistic model invoking repression or activation of one or two pathways did not explain the differential response to injury. Instead, our comparative transcriptomics showed a clear indication of fibrotic gene expression in *M. musculus*, while also revealing a unique transcriptional programme underlying regeneration in *A. cahirinus*. Hierarchical clustering highlighted six groups of genes, a total of 1,665 genes, with differential expression between species (Fig. 5b and Supplementary Data 1). Closer inspection of one cluster of genes that was upregulated in both species suggested a role for ECM organization in the injury response. To gain mechanistic insight into how the ECM might direct the injury response, we interrogated our data set to uncover transcriptional changes in the mammalian matrisome (see Methods). This approach classified a distinct subset of matrisome components with a pro-fibrotic or pro-regenerative signature. For example, we detected increased expression of collagen subunits and chondroitin sulfate proteoglycans (for example, *Fbn1*, *Col1a1*, *Col3a1*, *Lum* and so on) in *M. musculus* (relative to *A. cahirinus*) that are indicative of scarring (Fig. 5c)^6^,^7^. In contrast, we detected increased and sustained expression of molecules in *Acomys* (relative to *M. musculus*) suggesting peripheral nerve stimulation (for example, *Lgi2*, *Lgi3*, *Cthrc1*, *Lama1* and *Nell1*)^8^,^9^,^10^,^11^ and cell proliferation (for example, *Tnc* and *Fn1*) (ref. 12).

**Figure 6:**
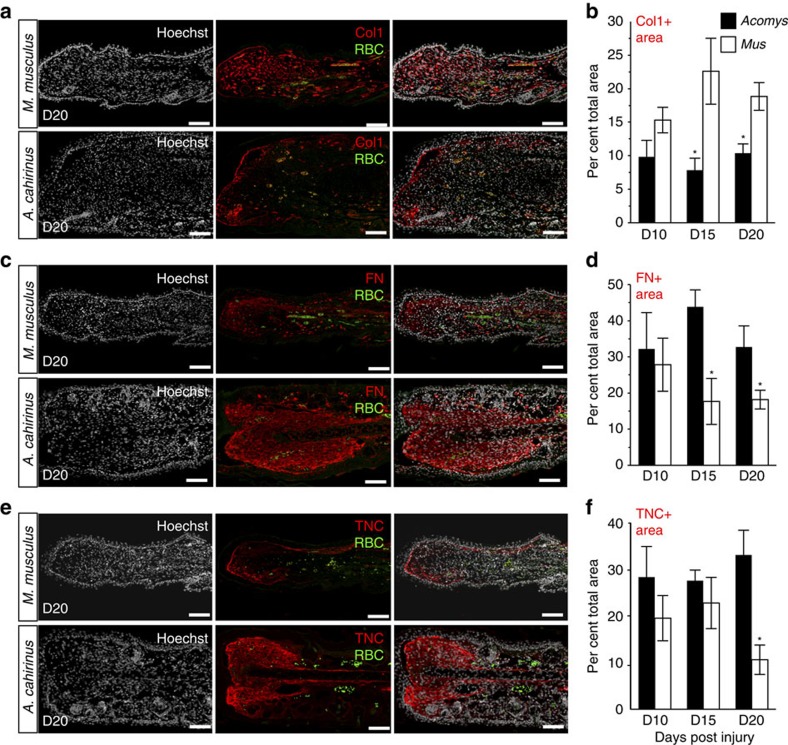
Immunostaining for the extracellular matrix proteins reveals differences in the total area of protein deposition. (**a**,**c**,**e**) Representative images for immunohistochemistry to detect collagen I (Col1) (**a**), fibronectin (FN) (**c**) and tenascin (TNC) (**e**) in *M. musculus* and *A. cahirinus*. Nuclei were counterstained with Hoechst (grey), antigen was detected by fluorescent secondary antibody (red), autofluorescent red blood cells (RBC) (green). (**b**,**d**,**f**) To quantify positive signal, a region of interest was created to include tissue distal to the original cartilage and to exclude hair follicles and epidermis. The ratio of total immuno-positive area per total area of the region of interest was calculated. Data represent mean and s.e.m., *t-test *P*<0.05 for *M. musculus* versus *A. cahirinus* comparison for the day indicated. One representative image per stain is shown; *n*=4 biological replicates per species per time point. Scale bar, 100 μm.

We performed quantitative PCR on a subset of ECM genes directing fibrosis and regeneration (*Col1a1*, *Col3a1*, *Tnc*, *Fn1*, *Mmp9* and *Mmp13*) and largely corroborated our RNA-Seq data (Fig. 5d). In *A. cahirinus*, we saw increased expression of *Mmp9*, *Mmp13*, *Fn1* and *Tnc*, presumably supporting a regenerative environment. In contrast, an intense fibrotic response in *M. musculus* was marked by an early and greater induction of *Col3a1* and *Col1a1* compared with *A. cahirinus.* Extending our gene expression analyses, we quantified protein distribution by immunohistochemistry in healing dermis at D10, D15 and D20 (Fig. 6a–f and Supplementary Fig. 5). We observed an extracellular matrix dominated by fibronectin and tenascin in the case of regeneration, and collagen 1 in the case of scarring (Fig. 6a–f and Supplementary Fig. 5). Together, our gene and protein data support induction, deposition and persistence of an extracellular environment that promotes regeneration in *A. cahirinus* and scarring in *M. musculus*.

### Spiny mice form a blastema during regeneration

A unifying feature of vertebrate appendage regeneration, when it occurs, is the formation of a blastema. Essentially, a blastema is a transient accumulation of lineage-restricted progenitor cells that proliferate and undergo organogenesis to regenerate the missing tissue (reviewed in ref. 13). The blastema is comprised of a specialized wound epidermis that attracts migrating cells and secretes factors that stimulate cell cycle progression and proliferation^14^,^15^. Blastema cells secrete, and are modified by, an ECM that is distinct and that promotes regeneration^12^. Finally, the blastema requires innervation to maintain the wound epidermis and promote cell proliferation (reviewed in ref. 16). To test if *A. cahirinus* formed a blastema during regeneration, we assessed healing tissue for the above characteristics.

First, we analysed the epidermis and observed a slower re-epithelialization rate in *A. cahirinus* compared with *M. musculus* (Supplementary Fig. 6a–c). In *M. musculus*, all animals re-epithelialized the cut surface by D10, with 80% re-epithelialized by D5 (Supplementary Fig. 6c). In contrast, only 50% of *A. cahirinus* re-epithelialized ear injuries by D10 (Supplementary Fig. 6c). Although fast re-epithelialization occurs in many non-amniotes, studies have shown that the speed of re-epithelialization is a not a pre-requisite for regeneration^17^, and a slow rate of re-epithelialization is consistent with mammalian digit-tip regeneration^18^. Instead, maturation of the neo-epidermis into an active wound epidermis is a key component of blastema formation. During salamander limb regeneration, the wound epidermis has distinct features including loss of keratinocyte apical–basal polarity, absence of a mature basement membrane and loss of a fully stratified phenotype^15^,^19^.

**Figure 7:**
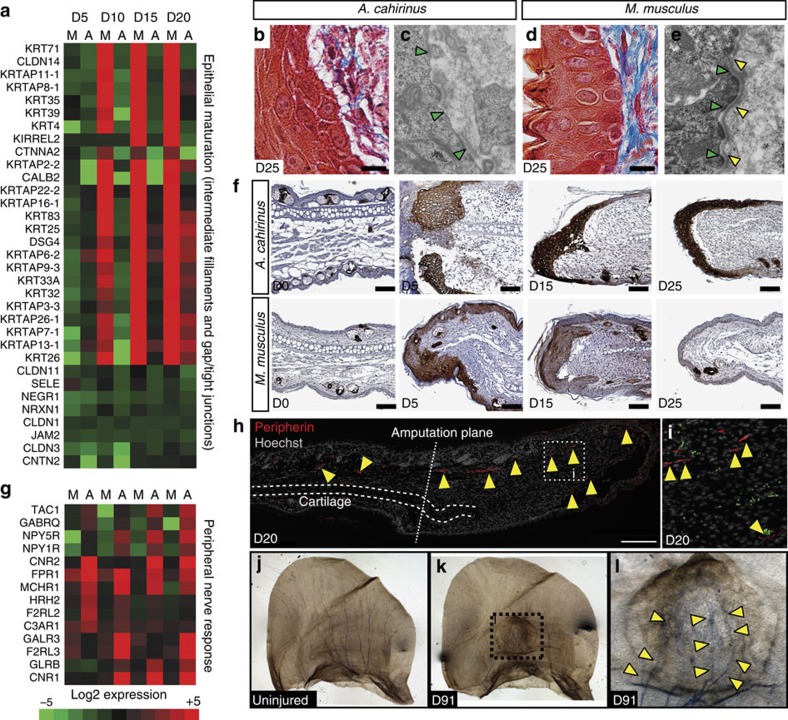
*A. cahirinus* forms an active wound epidermis and the blastemal is associated with peripheral nerve regeneration. (**a**) Heatmap showing genes with differential expression (increased in *M. musculus*, no change/decreased in *A. cahirinus*) associated with ‘intermediate filaments', ‘tight junctions' and ‘gap junctions'. Data represent log_2_(mean expression). (**b**,**d**) *A. cahirinus* (**b**) wound epidermis exhibits loss of apical–basal polarity in keratinocytes compared with *M. musculus* (**d**). (**c**,**e**) Electron micrographs comparing the wound epidermis in *A. cahirinus* (**c**) and *M. musculus* (**e**). The basement membrane in *M. musculus* shows both a lamina lucida (green arrows) and lamina densa (yellow arrows) (**e**), whereas only a lamina lucida is present in *A. cahirinus* (**c**). (**f**) Immunostaining for the basal keratinocyte marker, keratin 17 (dark brown), shows high abundance in *A. cahirinus* throughout the course of ear-hole closure. In *M. musculus,* keratin 17 was present in the epidermis at D5, became patchy at D15 and was absent from the epidermis at D25. Scale bar, 100 μm. (**g**) Heatmap showing genes with differential expression (decreased/no change in *M. musculus*, increased in *A. cahirinus*) associated with ‘neuroactive ligand receptor interaction' identified via ENRICHR. (**h**,**i**) Representative image showing immunohistrochemistry for peripherin (red) counterstained with Hoechst (grey) to show axon presence beyond the amputation plane at D20 in *A. cahirinus*. Arrows point to positive signal. Scale bar, 200 μM (**h**). (**j**–**l**) Representative images of Sihler's stained ears showing nerve tracts across the external ear in uninjured *A. cahirinus* tissue (**j**). D91 post-injury nerves regrow through the regenerated tissue (**k**). Higher-magnification image of boxed area in **k** showing nerve growth through regenerated tissue with higher density of nerve tracts at the proximal side of the injury (arrows) (**l**). All images are representative of at least *n*=3 biological replicates per species per time point.

Pathway analysis for a cluster within our RNA-Seq data set revealed increased differential expression of genes associated with epidermal maturation in *M. musculus* compared with *A. cahirinus* (Fig. 7a). Supporting the transcriptional response, we observed distinct differences in epidermal morphology between *A. cahirinus* and *M. musculus* (Fig. 7b,d and Supplementary Fig. 4a,b). While the neo-epidermis in *M. musculus* presented as a fully stratified squamous epithelium as early as D5, basal keratinocytes in *A. cahirinus* lacked apical–basal polarity and maintained cell–cell contact with the underlying mesenchymal cells during ear-hole closure (Fig. 7b,d). Transmission electron microscopy also revealed the lack of a mature bi-layered basement membrane and highlighted cell–cell connections present in the epidermis of *A. cahirinus,* but not in *M. musculus* at D25 (Fig. 7c,e). Another characteristic feature of the wound epidermis is the relative absence of proliferating cells^20^. Proliferating cells were found throughout the epidermis in *M. musculus* concomitant with early differentiation of the epidermis^1^, whereas actively proliferating cells are restricted to the proximal borders of the wound epidermis in *A. cahirinus* (Supplementary Fig. 6d–f). In addition to a distinct morphology and paucity of proliferating cells, several genetic markers of the wound epidermis have been identified in salamanders and newts^21^,^22^,^23^. The best characterized of these markers are keratins 5 and 17 whose expression is upregulated in the wound epidermis following amputation and persists through re-differentiation^21^. In response to injury, we found presence of keratin 17 throughout the neo-epidermis of *A. cahirinus* and *M. musculus* (Fig. 7f). In *M. musculus*, keratin 17 was present at D5, exhibited a patchy distribution by D15 and was completely absent at D25 except from hair follicles (Fig. 7f). In stark contrast, *A. cahirinus* exhibited intense keratin 17 staining in the wound epidermis throughout ear-hole closure (Fig. 7f). Taken together, our results demonstrate that a wound epidermis forms in *A. cahirinus*, similar to that seen in other vertebrate blastemas. However, whether or not a nascent wound epidermis forms in *M. musculus*, but does not mature, is unclear.

Another key feature of blastema formation is recruitment of mesenchymal cells to the injury site and modification of the extracellular environment to promote cell proliferation^15^. Initially, we observed mesenchymal cells accumulating beneath the epidermis in both *A. cahirinus* and *M. musculus* (Supplementary Fig. 4a,b). Examining healing tissue at D15 in *M. musculus*, the majority of cells exhibited classical spindle-shaped fibroblast morphology, whereas the majority of cells in *A. cahirinus* exhibited a rounded morphology reminiscent of fibroblasts found in other regenerating systems (Supplementary Fig. 7a,c). Analysing the subcellular structure of these cells, we noted active protein synthesis at the ribosomes in both species (Supplementary Fig. 7b,d). However, while fibroblasts in *M. musculus* contained nuclei with densely stained regions of heterochromatin, many fibroblasts in *A. cahirinus* exhibited a euchromatic nucleus (Supplementary Fig. 7b,d).

Re-innervation of the blastema is required during epimorphic regeneration. Analysis of our RNA-Seq data identified genes associated with axon guidance, neuroactivity and growth, which were enriched in *A. cahirinus* compared with *M. musculus* (Fig. 7g). To assay for re-innervation, we labelled axons during regeneration using peripherin, a neural intermediate filament that provides structure to axons. At D10, peripheral nerve endings were identified distal to the amputation plane (Supplementary Fig. 8), and at D20, there was clear evidence for re-innervation of the blastema (Fig. 7h,i). Sihler staining revealed mylenated axons fanning out across the uninjured ear pinna (Fig. 7j) and showed axonal extensions throughout the regrown tissue from the proximal portion of the injury at D91 (Fig. 7k,l). Interestingly, axons were absent from the distal-most part of the new tissue suggesting that the proximal/distal bias in growth we observed during regeneration could be attributed in part to a lack of innervation. Taken together, these data demonstrate cellular and molecular attributes of blastema formation in *A. cahirinus*.

### Blastemal cells exhibit cell cycle progression and division

**Figure 8:**
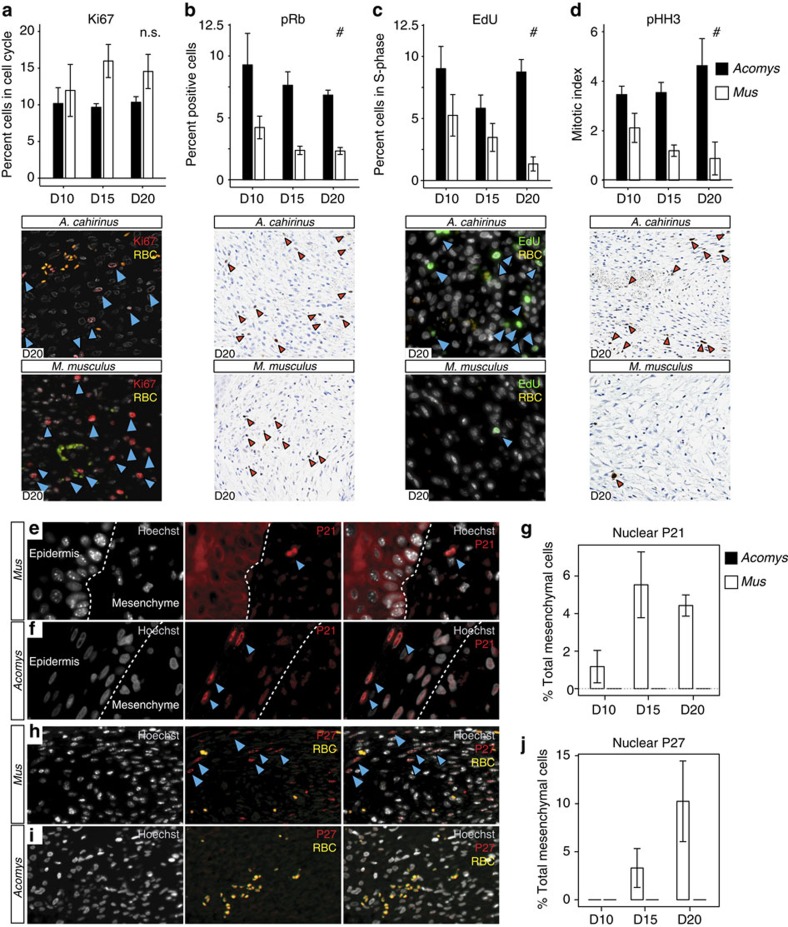
Nuclear localization of p21 and p27 is coincident with a lack of cell cycle progression in *M. musculus*. (**a**–**d**) Per cent positive cells (of total counted) for cell cycle markers (**a**) Ki67—cell cycle re-entry, (**b**) pRb—G1/S transition, (**c**) EdU—active DNA synthesis and (**d**) pHH3—mitotic cells (blue and red arrows). (**a**) Per cent Ki67+ mesenchymal cells are not different between *M. musculus* and *A. cahirinus* at D10, D15 and D20 (*n*=5 biological replicates for all time points except D10 and D15, *A. cahirinus n*=4). (**b**) Per cent pRb+ mesenchymal cells are greater in *A. cahirinus* at all time points (*n*=3 biological replicates for both species at each time point). (**c**) Active DNA synthesis is maintained during regeneration in *A. cahirinus*, whereas the number of EdU+ cells in *M. musculus* continually declines to <1% at D20 (D10, *n*=5 both species; D15, *A. cahrinus n*=5, *M. musculus n*=2; D20, *A. cahirinus n*=3, *M. musculus n*=3). (**d**) Per cent pHH3+ cells increase in *A. cahirinus* during regeneration, while pHH3+ cells decline in *M. musculus* with few detected at D20 (*n*=3 for all time points except D20, *M. musculus n*=2). Data represented as mean and s.e.m., NS (not significant) and # denote *P*>0.05 or *P*<0.05, respectively, for one-way ANOVA for species effect; *P*>0.05 for all comparisons for effect of day (**a**–**d**). (**e**,**f**) Nuclear localization of p21 is coincident with lack of cell cycle progression. (**e**) Cytoplasmic p21 localization corresponds to highly proliferative epidermal cells in *M. musculus,* whereas nuclear staining is observed in mesenchymal cells at D15 (**f**). The reverse is true in *A. cahirinus.* Nuclear localization is evident in epidermal cells of the non-proliferative wound epidermis, while mesenchymal cells do not exhibit nuclear staining. (**g**) Per cent total p21 positive nuclei in the mesenchyme of *M. musculus* and *A. cahirinus* (*n*=3 for all time points). (**h**) Immunostaining for p27 shows positive nuclear staining in *M. musculus* mesenchymal cells (**i**). Cells in the blastemal region of *A. cahirinus* are negative for nuclear p27. (**j**) Quantification of per cent total p27 nuclear staining in the mesenchyme of *A. cahirinus* and *M. musculus* (*n*=3 biological replicates for all time points). See Supplementary Fig. 9 for positive controls (**f**,**i**).

The defining feature of a blastema is that accumulating cells re-enter the cell cycle, proliferate and continue to divide throughout the course of regeneration. Therefore, we quantified the fraction of cells that re-entered the cell cycle (that is, those with proliferative potential) in response to injury in *A. cahirinus* and *M. musculus*. Using Ki67 to mark cells in G1-, S- and G2/M-phase, but not those that are in G0 (resting or quiescent), we found positive cells in both species (Fig. 8a). Quantifying Ki67+ mesenchymal cells, we found no significant difference in the percentage of cycling cells across time points between species (two-way ANOVA, *F*=1.18, *P*=0.3527; Fig. 8a). Although Ki67+ cells indicate cell cycle re-entry, numerous cell cycle checkpoints guard against unregulated cell cycle progression, and arrested cells can remain Ki67+ (ref. 24). Therefore, we next asked if mesenchymal cells from both species were actively progressing through the cell cycle and dividing using stage-specific markers. Phosphorylation of retinoblastoma (pRb) protein facilitates the G1/S transition by releasing bound E2F1 to activate target genes and push cells into S-phase^25^. Quantifying pRb+ cells, there was no species × day interaction (two-way ANOVA, *F*=0.30, *P*=0.7460) and we found significantly more pRB+ cells in *A. cahirinus* compared with *M. musculus* at all time points (one-way ANOVA, *F*=37.21, *P*<0.0001; Fig. 8b). This result indicated that a significantly higher proportion of cells in *A. cahirinus* were actively moving into S-phase compared with *M. musculus*. To quantify cells in S-phase that were actively replicating DNA, we administered a short pulse of EdU 30 min before tissue collection. As with pRb+ cells, there was no species × day interaction (two-way ANOVA, *F*=1.84, *P*=0.1884) and we detected significantly more EdU+ cells in *A. cahirinus* compared with *M. musculus* at all time points (one-way ANOVA, *F*=14.17, *P*=0.0015; Fig. 8c). Thus, while we detected very few EdU+ positive cells in *M. musculus* at D20, a significant fraction of blastemal cells in *A. cahirinus* were actively undergoing DNA synthesis (Fig. 8c). To complete cell division, cells must transition from S-phase to G2/M and undergo mitosis. Quantifying phosphorylated histone H3 (PHH3) to label cell mitotic cells, there was no species × day interaction (Two-way ANOVA, F=2.60, p=0.1186) and we detected significantly more PHH3+ cells in *A. cahirinus* at every time point compared to *M. musculus* (one-way ANOVA, F=29.07, *P*=0.0002) (Fig. 8d). Moreover, very few PHH3+ cells were detected in *M. musculus* at D15 or D20 (Fig. 8d). Together, these results demonstrate that mesenchymal cells in *A. cahirinus* re-enter the cell cycle, proliferate and divide. In contrast, although cells in *M. musculus* re-enter the cell cycle, our results suggest that most cells fail to exit G1 or progress through S-phase, and the small amount of cell division observed is not sustained. Importantly, our finding that mesenchymal cells actively divide and proliferate in association with innervation and wound epidermis formation further support the conclusion that a blastema forms in *A. cahirinus*.

### Nuclear localization of p21 and p27 during scarring

Reduced cell cycle progression of mesenchymal cells in *M. musculus* prompted us to examine possible causes for cell cycle arrest at the G1–S checkpoint. *In vitro* data comparing newt and mammalian myotubes suggest cells in non-regenerative tissue fail to phosphorylate pRb and enter S-phase^26^, and *in vivo* studies in mammals suggest a role for the cyclin-dependent kinase inhibitors, p21 and p27, during cellular senescence^27^,^28^. The ability of cell cycle regulators to control cell cycle progression rests largely on their cellular localization (reviewed in ref. 29). Specifically, nuclear localization of p21 and p27 inhibit cell division^30^, whereas their cytoplasmic localization can initiate G1/S progression as chaperones for cyclin E (refs 31, 32). Analysing the cellular localization of p21 and p27 in *A. cahirinus* and *M. musculus* revealed striking differences (Fig. 8e–j). First, comparing the wound epidermis in *A. cahirinus* (non-proliferative) to the distal epidermis in *M. musculus* (proliferative), we found nuclear localization of p21 and cytoplasmic localization, respectively (Fig. 8e,f). When analysing mesenchyme beneath the epidermis we detected strong nuclear localization in *M. musculus* cells, but were unable to detect nuclear localized p21 in *A. cahirinus* cells (Fig. 8e,f). Moreover, the number of mesenchymal cells positive for nuclear localized p21 in *M. musculus* increased over time (Fig. 8g). Although we did not detect nuclear localized p21 in *A. cahirinus* blastemal cells, we did detect positive cells proximal to the injury (Supplementary Fig. 9a–c). We next quantified the number of mesenchymal cells that were positive for p27 (Fig. 8h–j). Similarly, while we detected almost no p27+ cells in *A. cahirinus*, the percentage of p27+ mesenchymal cells in *M. musculus* increased at D15 and D20. As with p21, while we did not detect p27+ blastemal cells beneath the wound epidermis in *A. cahirinus*, we detected many p27+ cells proximal to the injury site in cartilage, perichondrium and associated with skeletal muscle (Supplementary Fig. 9d–j). These data suggest that surveillance of injury-induced cell cycle re-entry in *M. musculus* leads to the production and shuttling of p21 and p27 to the nucleus where they inhibit cell cycle progression.

## Discussion

Studying adult mammals capable of *bonafide* tissue regeneration has important implications for understanding the evolution of regenerative mechanisms in vertebrates and for advancing clinical regenerative medicine. Paramount to this task is reliably identifying instances of epimorphic regeneration and distinguishing them from the normal range of wound-healing abilities. To this end, our results support the need to quantify ear closure pattern and couple this with cellular analysis of healing tissue to demonstrate tissue regeneration. Using this combined assay we discovered that *A. cahirinus* can regenerate skin, sebaceous glands, hair follicles, adipose tissue and cartilage, and confirmed that *O. cuniculus* rapidly regenerates ear tissues as well^2^,^3^. In a phylogenetic context, whether or not ear pinna regeneration extends to other members of the Deomyinae (for example, Lophuromys, Deomys and Uranomys) remains untested. The only other mammal species for which ear tissue regeneration has been reliably reported are hares, pikas and cats^33^, although a lack of data makes these reports in need of replication. Our data for *M. musculus* strains (CD1, Swiss Webster and MRL/MpJ) and *M. brockmani* coupled with published reports showing no regeneration in other mammals^33^,^34^, support the conclusion that epimorphic regeneration among mammals is quite rare. Although broader sampling is required, the available data suggest enhanced regenerative ability has evolved independently at least several times in Eutherian mammals^35^,^36^ and it will be interesting to investigate the molecular regulation of these processes across species.

In addition to our analyses of regenerative ability, our statistical model demonstrated that biotic factors could affect ear closure rate. In other regenerating vertebrate systems, fundamental traits such as age, sex and metamorphosis affect regeneration rate, not the ability to mount a regenerative response^37^,^38^,^39^,^40^,^41^. Our data from *Acomys* support these observations. In line with previous work in rabbits, we found intraspecific variation in regeneration rate based on sex in *A. kempi* and *A. cahirinus*^37^,^42^. Interestingly, it only manifested following blood collection in *A. cahirinus*. While it is difficult to know why blood collection caused the observed sex bias in regeneration rate, the additional injury clearly caused a difference in the physiological response between male and female spiny mice that could be explained by differences in immunomodulation. Human studies examining inflammation in response to injury have shown that as the severity of injury increases, females have a greater pro-inflammatory response compared with males^43^,^44^,^45^. A similar response may be operating in spiny mice and if so, suggests a stronger pro-inflammatory response might accelerate regeneration rate.

Our cellular analysis showed that complete ear-hole closure was coupled with tissue regeneration and restoration of the original tissue architecture. In contrast, partial ear-hole closure in non-regenerating species was coupled with scar tissue accumulation. Using multiple size holes to analyse regeneration, we found that wound size did not appear to limit regenerative ability in regenerators. Our cellular analysis of 4-mm holes demonstrates that MRL/MpJ mice heal these injuries with scarring rather than by regenerating tissue. These results are in line with a number of robust studies showing that MRL/MpJ mice exhibit intense fibrosis in response to skin^46^ and heart injuries^47^,^48^. Importantly, the results of this study demonstrate that regeneration in *A. cahirinus* is epimorphic (that is, cell proliferation before differentiation) and proceeds through blastema formation. Our detailed comparative analysis suggest that four major requirements for a mammalian blastema are the following: (1) the ability to form and sustain a wound epidermis characterized by basal keratin markers, absence of proliferating cells and a secretory phenotype; (2) activation and maintenance of a pro-regenerative ECM; (3) an accumulation of mesenchymal cells that maintain cell division through multiple rounds of mitosis; and (4) re-innervation. A regenerative wound epidermis is required for appendage regeneration (reviewed in ref. 49), and interactions between the epidermis and underlying mesenchyme control the balance between cell division and differentiation^14^. In addition to key morphological features, we demonstrated that persistent keratin 17 expression labels the mammalian wound epidermis. In addition, loss of keratin 17 expression in *M. musculus* corresponds to the formation of a fully stratified squamous epithelium. As mesenchymal cells accumulated beneath the wound epidermis, expression profiles for ECM genes indicated a pro-regenerative environment^1^,^12^,^17^,^50^,^51^. This gene expression data support our previous observation of a qualitative bias towards an ECM rich in tenascin-C and poor in collagen type 1 in spiny mice^1^. Our present data also suggest the matrix remodelling enzymes MMP9 and MMP13 further antagonize collagen aggregation, which is antagonistic to regeneration. Both enzymes are essential for appendage regeneration in axolotl and newt limbs^52^,^53^.

Our comparative analysis demonstrated that the molecular regulation of regeneration is complex, and not determined by differential regulation of one or two genes or pathways. Our gene and protein data emphasize that precise control and induction of specific set of ECM genes is at least partly responsible for causing a regenerative response. The comparative analysis showed that mesenchymal cells from *A. cahirinus* and *M. musculus* re-entered the cell cycle in response to injury. We also detected a small fraction of *M. musculus* cells that were mitotic suggesting a small burst of proliferation. However, sustained cell cycle progression and cell division was only observed in *A. cahirinus*. Instead, nuclear localization of the cell cycle regulators p21 and p27 corresponded to a maturation of the epidermis, cessation of ear closure and loss of cell cycle markers other than Ki67. The nuclear localization of p21 and p27 was not observed in *A. cahirinus* blastema cells even though positive cells were observed in uninjured tissues proximal to the injury site. The involvement of p21 and p27 to inhibit cell cycle progression suggests the activation of cellular senescence in response to injury^27^,^28^. Understanding how these and other tumour suppressors function in response to injury may be a key component separating regeneration from scarring^54^.

Another unifying feature of vertebrate regeneration is that lineage-restricted progenitor cells create new tissue *de novo* from a mass of blastema cells rather than directly from existing tissue^55^,^56^,^57^,^58^, as occurs during fracture healing or spike regeneration in *Xenopus* froglets^59^,^60^. Our finding that some new cartilage formed directly from existing cartilage in non-regenerating species suggests that perichondrial cells might contribute progeny that directly form additional cartilage nodules, although the cellular source of these nodules remains unknown. In regenerating species, the overall amount of regrown cartilage and new condensations located far from cut cartilage suggests an additional cellular source, possibly from de-differentiated fibroblasts. In addition, removal of the wound epidermis leads to precocial differentiation of cartilage^14^ and thus, cartilage nodules in non-regenerating species may represent the early burst of proliferation observed in *M. musculus* that is lost on maturation of the neo-epidermis. These findings underscore the importance of how specific cell lineages respond to injury and support the fibroblast lineage as a key cell type during complex tissue regeneration. Recent studies suggest that fibroblast populations are heterogeneous and indicate that specific populations of fibroblasts may bias wound-healing outcomes in mammals^61^,^62^. The data presented here showed that the fibroblasts in the ear pinna of *A. cahirinus* and *M. musculus* responded differently in the 4-mm ear punch assay. It is possible that identifying the mechanistic underpinnings of the regenerative fibroblast response may be instructive towards reducing fibrosis in a non-regenerating species.

While regeneration researchers have historically drawn a firm distinction between epimorphic regeneration and wound healing, the increasing interest in regenerative medicine has created the concept that mammalian tissue regeneration is one extreme along a healing continuum with scarring at one end and regeneration at the other. Our data show that tissue regeneration in the ear pinna is distinct from wound healing because the ability to regenerate does not appear variable: all regenerating species closed their ear holes and all non-regenerating species failed to close ear holes. This unites regeneration in spiny mice and rabbits with regeneration in other vertebrates such as salamanders, newts and zebrafish, where all healthy adults regenerate in response to injury. This finding provides an experimentally supported paradigm for researchers using mammals to interpret how various organ systems respond to injury. Future work analysing regenerative ability across a broad phylogenetic spectrum should provide insight into core cellular and molecular features that may be hallmarks of a mammalian regenerative response.

## Methods

### Animals, husbandry and ethics

*A. cahirinus* and CD1 mice (*M. musculus*) were housed at the University of Kentucky, Lexington, KY. *A. cahirinus* were housed at a density of 10–15 individuals in metal wire cages (24 inch × 18 inch × 16 inch, height × width × depth; Quality Cage Company, Portland, OR) and fed a 3:1 mixture by volume of 14% protein mouse chow (Teklad Global 2014, Harlan Laboratories, Indianapolis, IN) and black-oil sunflower seeds (Pennington Seed Inc., Madison, GA) 1 × per day. CD1 mice were fed mouse chow only. *A. cahirinus* and CD1 mice were kept on a 14:10 (light:dark) light cycle. MRL/MpJ mice were a gift to A.W.S. from Edward Scott (University of Florida), and the experiments were completed at the University of Florida. MRL/MpJ mice were housed in individually ventilated cages, fed 18% protein mouse chow (Teklad Global 2918) and kept on a 12:12 (light:dark) light cycle. All animals used were sexually mature and sexes can be found in Supplementary Table 1.

*A. kempi* and *M. brockmani* were live-captured in Laikipia, Kenya, and transported to the University of Nairobi for study. Swiss Webster mice (*M. musculus*) and New Zealand white rabbits (*O. cuniculus*) were obtained from local breeders in Nairobi, Kenya. Each rodent species was separated by sex and housed at a density of 10–15 animals in metal wire cages (Quality Cage Company), fed mouse pencils (Argrocide Inc., Nairobi, Kenya) 1 × per day and exposed to natural light through windows (equivalent 12:12 light:dark cycle). The rabbits were housed at a density five rabbits per pen, exposed to natural light through windows and given rabbit chow (Agrocide Inc.) 1 × per day supplemented with fresh carrots and kale greens.

All animal procedures were approved by the University of Kentucky Institutional Animal Care and Use Committee (IACUC) under protocol 2013-1119, Kenyan Wildlife Service (KWS) and the University of Nairobi Faculty of Veterinary Medicine Animal Care and Use Committee (FVM ACUC). Research in Kenya was approved by the Kenyan National Council for Science and Technology (NACOSTI). All wild species trapped were species of least concern.

### Species identification

Species were identified in the field using gross morphology, body colour and body measurements. To further confirm species identity, a ∼750-bp fragment of mitochondrially encoded cytochrome c oxidase I was PCR amplified using the universal primers BatL5310 (5′-CCTACTCRGCCATTTTACCTATG-3′) and R6036R (5′-ACTTCTGGGTGTCCAAAGAATCA-3′) and sequenced from five individuals of each species. These sequences were trimmed to 600 bp and input into the BOLD Systems database (http://www.boldsystems.org/) for identification. A result of ≥99% nucleotide identity was used as a positive species identity.

### 4-mm ear punch assay

Animals were anaesthetized with 4% (v/v) vaporized isoflurane (Henry Schein Animal Health, Dublin, OH) and subjected to a through-and-through hole created in the right and left ear pinna using a 4-mm biopsy punch (Sklar Instruments, West Chester, PA). In all rodents the ear punch was placed ∼1 mm distal from the head and centred on the middle of the pinna. For rabbits, the punch was placed approximately halfway up the ear between the central vein and artery. Following injury, animals were anaesthetized every 5 days (or until D30 and then at D40, D60 and D85, if the hole was no longer closing) and calipers were used to measure the diameter along the proximal–distal (*D*_PD_) and anterior–posterior (*D*_AP_) axes for each ear hole. D85 was the end of the experiment for all animals in Kenya and most in Kentucky. Ear-hole area was calculated for an ellipse to account for any unevenness in closure along either axis (equation (1)). An ear hole with diameter measurements <0.5 mm was denoted a pinhole and given a value of zero. When no light could be seen through the hole in the ear the hole was considered closed.


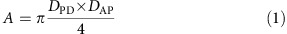


### Blood collection

Blood was collected from a subset of animals (Supplementary Table 1) via the submandibular venous bed on D0, D1 and D15. Briefly, individuals were anaesthetized with 4% (v/v) isoflurane and gently scruffed so that the skin covering the submandibular venous bed was taut. Next, a 5-mm lancet (Medipont Inc., Mineola, NY) was inserted quickly into the venous bed and 3–4 drops of blood were collected into a 0.5-ml microcentrifuge tube. The volume of blood removed was approximately equal to 0.3–0.7% of total blood volume.

### Pregnancy determination

Four groups of female *A. cahirinus* were used to determine if pregnancy or lactation affects ear-hole closure. Group A consisted of virgin females experiencing their first pregnancy. Virgin female *A. cahirinus* between 8 and 15 weeks old were mated with males known to be fertile. Each subsequent morning, females were observed for the presence of a vaginal plug. Because plugs are not visible in *A. cahirinus*^63^ females were palpitated for fetuses each morning after being with a male for 10 days. The 4-mm ear punch assay was performed between D15 and D20 of pregnancy. The approximate developmental day of pregnancy when the ear-hole punch was performed was determined from the day of birth based on a 39-day gestation^64^. Group B consisted of non-pregnant, control females from group A. Group C leveraged post-partum oestrus to test pregnancy and lactation^65^. Females deemed pregnant through palpitation were placed with males before giving birth. The day of birth designated day 1 of pregnancy and a 4-mm punch was made on day 15. Only females that gave birth to a litter were used in this group. Group D were dams that did get pregnant from group C and represented non-pregnant, lactating females.

### Statistics

To understand the kinetics of ear-hole closure, we first graphed the mean ear-hole size versus day post injury for each species × sex combination. The data used were hole area from one ear (left or right) per individual that did not tear or was collected for histology throughout the entire experiment, or the averaged hole area from both ears if both ears followed the above criteria. Ear holes from animals that died or appeared sick during the experiment were excluded. To model the ear-hole area of each mouse, a repeated measures ANOVA was fit to area using species, sex, a polynomial effect of time, and all interaction effects. The polynomial time effect is used to search for linear (*y*=*x*^1^), quadratic (*y*=*x*^2^), cubic (*y*=*x*^3^) and quartic (*y*=*x*^4^) curves and so on. The repeated measures ANOVA with a polynomial time effect identified a cubic time effect from the data. To make intra- and interspecific comparisons, we used a stratified analysis within each species and sex, respectively. Some comparisons and tests of effects used adjusted *P* values for the test since unadjusted *P* values were not necessarily valid. These cases are noted in the text and tables. In all tests, significant differences were defined to be when the *P* value was <0.05. Day 0 was not included in the model due to the lack of variation in ear-hole area at the start of the experiment. To avoid violation of model assumptions, the final day for the model was determined as the last measurement day before >80% of observations were equal to zero, again because of the lack of variation. Thus, because *A. cahirinus* had the fastest closure (>80% closed by D35) of the included species, we used D5–D30 for all species.

Determination of ear-hole measurement variation was completed first on a subset of the animals in the data (*A. cahirinus* and CD1 *M. musculus*) in Kentucky. No deliberate randomization methods were used; however, the animals included all analyses appeared healthy with no overt problems.

For analysing cell cycle markers, observations within the same mouse were averaged so analysis could be on the level of the experimental unit. For each marker, we analysed the percentage of labelled cells as a fraction of total cell number using a two-way ANOVA with species, day and the species × day interaction. The percentages were transformed before analysis by taking the square root, followed by the arcsine of each value. In addition, the data were assumed to be normal for this analysis. All statistics were performed using SAS (SAS Institute Inc., Cary, NC). Graphs were created using R (R Core Team, Vienna, Austria) or Prism (GraphPad Software Inc., La Jolla, CA) and annotated within Illustrator (Adobe).

### Tissue preparation and histology

Tissue was collected at the end of the experiment from a subset of injured ears using an 8-mm punch biopsy at D85 (or D96). Collected tissue was placed into 10% (v/v) neutral buffered formalin (American Master Tech Scientific Inc., Lodi, CA) and incubated at 4 °C overnight with agitation. Tissue was washed three times with PBS, three times with 70% (v/v) ethanol and stored at 4 °C in 70% (v/v) ethanol. All tissue processing was done at the University of Kentucky using a rapid microwave histoprocessor (Micron Instruments, Inc. Carlsbad, CA). Tissue was embedded in paraffin (Leica Biosystems, Buffalo Grove, IL) and 5-μm sections were placed onto Superfrost Plus slides (Fisher Scientific). Tissue sections were processed for routine histology and stained with Masson's trichrome (Richard-Allen Scientific, Kalamazoo, MI).

For subcellular analysis, D15 ear tissue was collected and used for the routine preparation of transmission electron microscopy under supervision from the University of Kentucky Imaging Facility. Briefly, wound bed tissue was diced and fixed in a droplet of freshly prepared 4% (w/v) paraformaldehyde/3.5% (v/v) glutaraldehyde in 0.1 M cacodylate buffer (PFA/GLA), and further fixed by immersion in PFA/GLA for 2 h at 4 °C. After the first fixation, tissue pieces were washed four times with 8% (w/v) sucrose before post-fixing with 1% (w/v) OsO_4_ for 1.5 h at 4 °C. After dehydration through ethanol into propylene oxide, tissues were infiltrated and embedded into Eponate 12 (PELCO). Embedded tissue pieces were sectioned to ultrathin slices of about 70 nm using a Reichert Ultracut E, mounted on copper grids and stained with uranyl acetate followed by lead citrate.

To observe mature, myelinated nerve fibres in the ear we used Sihler's stain on whole ears^66^. The entire ear was collected from the animal and fixed in 10% (v/v) un-neutralized formalin for 7 days at room temperature with agitation. After washing in water for 1 h, the fixed tissue was macerated and depigmented with 3% (w/v) KOH with three drops of H_2_O_2_ until translucent (about 7 days). The depigmented tissue was washed in water for 1 h and then incubated in Sihler's solution I (1 glacial acetic acid:1 glycerine:6 1% (w/v) chloral hydrate) until decalcified or about 7 days. After washing in water for 1 h, the processed tissue was then stained in Sihler's solution II (1 Ehrlich's haematoxylin:1 glycerine:6 1% (w/v) chloral hydrate) until all of the nerves were stained deep purple or about 10 days. After washing for 1 h, the stained tissue was destained for about 2 h by incubating in Sihler's solution I. After destaining, the tissue was washed in water for 1 h and then neutralized by incubating with freshly made 0.05% (w/v) lithium carbonate for 1 h. Once stained, the tissue was cleared in 50% (v/v) aqueous glycerine for 5 days and then changed to 100% glycerine for 5 days before imaging.

### Immunohistochemistry

Immunohistochemical and immunofluorescent staining was performed on deparaffinized and rehydrated sections with specific primary antibodies (Supplementary Table 5) and detected with either Alexa Fluor-conjugated streptavidin or horseradish peroxidase-conjugated streptavidin for 3, 3′-diaminobenzidine (DAB) conversion (Invitrogen, Carlsbad, CA). Nuclei were counterstained with either 10 μg ml^−1^ Hoechst for fluorescence or Mayer's haematoxylin for bright-field visualization. Coverslips were mounted using either ProLong Gold mounting medium (Invitrogen, Carlsbad, CA) for fluorescence or XySeal for bright-field visualization. Total cell number was quantified using the ITCN plugin for ImageJ. To detect cells in S-phase, 100 μg EdU was intraperitoneally injected into *M. musculus* and *A. cahirinus* 30 min before tissue collection. Detection was carried out on formalin-fixed, paraffin-embedded tissue sections using the Click-it chemistry^67^.

To quantitatively determine the total area of positive signal, photomicrophages of entire representative digit sections were obtained at × 10 magnification using an Olympus BX53 fluorescent deconvolution microscope (Olympus America Inc). Quantification of positive signal was performed on four separate samples per time point by thresholding fluorescent signal and mask subsampling with Metamorph Imaging software (Molecular Devices). The ratio of total immuno-positive area per total area of the region of interest was then calculated. The ID of the sample being run was blinded from the user until the end of the analysis.

### Microscopy and image acquisition

Bright-field images of histology were taken on a BX53 light microscope (Olympus, Tokyo, Japan) using a charge-coupled device camera (Olympus). To obtain representative images across several fields, a digital stitching tool (Cell Sens, Olympus) was used and images were assembled using Photoshop (Adobe Systems Inc., San Jose, CA). Subcellular images were taken using a Tecnai Biotwin 12 transmission electron microscope (FEI, Hillsboro, OR) equipped with a charge-coupled device camera (FEI). Tissue from a minimum of three individuals was observed to make conclusions. All representative images in figures represent one of at least *n*=3 biological replicates that were observed, unless otherwise noted in figure legends.

### Transcriptomics

A ring of ear tissue ∼1 mm proximal from the healing margin was collected and flash-frozen from sexually mature female *A. cahirinus* and *M. musculus* at D0, D5, D10, D15 and D20 after the 4-mm punch assay. Total RNA was extracted using Trizol (Life Technologies) as per the manufacturer's protocol. Before sequencing, RNA integrity and concentration was measured using a bioanalyser (Agilent Technologies, Santa Clara, CA). One microgram of total RNA was used for the preparation of cDNA and barcoded strand-specific libraries, followed by high-throughput sequencing (Illumina 50-bp paired-end sequences). A total of 30 samples (5 time points × 3 biological replicates per time point × 2 species) were sequenced on three lanes as a barcoded pool. Sequencing data were analysed as follows. First, a *de novo* transcriptome was assembled for *A. cahirinus* using Trinity 2.1.1 (ref. 68). Second, RSEM 1.2.19 (ref. 69) and Bowtie 1.1.1 (ref. 70) were used to generate estimates of gene expression by mapping reads from individuals at each time point to their respective species-specific reference. The *A. cahirinus* reference was built from the *de novo* assembled transcriptome and the *M. musculus* reference was built from the mm10 gene annotations (http://hgdownload.soe.ucsc.edu/downloads.html#mouse). Third, EBseq^71^ was used to calculate fold-changes independently for each species to test for differential gene expression with respect to D0 using an false discovery rate (FDR)=0.05. Last, hierarchical clustering on gene expression data was performed using JMP 11 (ref. 72), to generate dendrograms and heatmaps of the ear transcriptome and matrisome. ENRICHR was used to determine gene ontologies for identified gene clusters^73^. The results from our high-throughput sequencing has been deposited in NCBI (GSE71761) and our EBseq results are summarized in Supplemental Data. To further explore genes that make up the extracellular matrix we made use of the validated list of ECM components comprising the ‘matrisome' (http://matrisomeproject.mit.edu).

To validate our expression data, we performed SYBR-green quantitative PCR (Quanta Biosciences, Gaithersburg, MD) with species-specific primers (Supplementary Table 4) and the cDNA libraries used for RNA-Seq on a Roche Lightcycler 96 (Roche Diagnostics, Indianapolis, IN). Primers were designed to bind in orthologous region of each gene in both species (for example, spanning orthologous exons with similar GC content and PCR product size) using the NIH Primer-Blast tool^74^. Results were analysed using ΔΔC_t_ with *Tbp* as the endogenous control relative to D0 calculated separately for each species.

### Code availability

The code specific to our ear-hole model conducted in SAS can be found on the corresponding author's website (www.ashleyseifert.com) and at Dryad.

## Additional information

**Accession codes:** Sequencing data have been deposited in the NCBI database under accession code GSE71761.

**How to cite this article:** Gawriluk, T. R. *et al*. Comparative analysis of ear-hole closure identifies epimorphic regeneration as a discrete trait in mammals. *Nat. Commun.* 7:11164 doi: 10.1038/ncomms11164 (2016).

## Supplementary Material

Supplementary Information Supplementary Figures 1-9 and Supplementary Tables 1-5

Supplementary Data 1 RNA-seq data. Gene expression changes relative to D0 for Acomys and Mus.

Peer Review file

## Figures and Tables

**Table 1:** Repeated measures ANOVA of ear-hole area over time.

**Source**	**DF**	**Type III SS**	**Mean square**	***F*****-value**	***P*** **value**
Day	5	1,127.936074	225.587215	95.17	<0.0001
Day × species	20	843.263122	42.163156	17.79	<0.0001
Day × sex	5	37.512270	7.502454	3.17	0.0206
Day × species × sex	20	119.952960	5.997648	2.53	0.0023
Error (day)	540	1,280.000720	2.370372		

DF, degrees of freedom; SS, sum of squares. There was a significant effect of time (day) on ear-hole area, and ear-hole area varied by species and species × sex. The *P* values are the H–F–L adjusted *P* values.

**Table 2:** Variation in ear-hole closure rate for pairs of species (males and females separated).

**Sex**	**Contrast**	**DF**	**Contrast SS**	**Mean square**	***F*****-value**	**Adjusted** ***P*** **value (H**–**F**–**L)**
Male	Day × ach versus ake	5	75.5500819	15.1100164	7.97	<0.0001
	Day × ach versus cd1	5	252.3020766	50.4604153	26.62	<0.0001
	Day × ach versus myo	5	39.5896515	7.9179303	4.18	0.0052
	Day × ach versus swi	5	117.1200211	23.4240042	12.36	<0.0001
	Day × ake versus cd1	5	36.7674594	7.3534919	3.88	0.0079
	Day × ake versus myo	5	21.5668797	4.3133759	2.28	0.0747
	Day × ake versus swi	5	41.4337384	8.2867477	4.37	0.0040
	Day × cd1 versus myo	5	23.5113021	4.7022604	2.48	0.0563
	Day × cd1 versus swi	5	46.5827196	9.3165439	4.92	0.0019
	Day × myo versus swi	5	19.2856559	3.8571312	2.04	0.1039
Female	Day × ach versus ake	5	23.9411171	4.7882234	1.83	0.1365
	Day × ach versus cd1	5	618.5524989	123.7104998	47.26	<0.0001
	Day × ach versus myo	5	65.6868979	13.1373796	5.02	0.0015
	Day × ach versus swi	5	102.4565207	20.4913041	7.83	<0.0001
	Day × ake versus cd1	5	409.0505056	81.8101011	31.25	<0.0001
	Day × ake versus myo	5	57.3966044	11.4793209	4.38	0.0037
	Day × ake versus swi	5	73.0942481	14.6188496	5.58	0.0007
	Day × cd1 versus myo	5	31.9735201	6.3947040	2.44	0.0588
	Day × cd1 versus swi	5	134.0643634	26.8128727	10.24	<0.0001
	Day × myo versus swi	5	22.3392778	4.4678556	1.71	0.1609

ach, *A. cahirinus*; ake, *A. kempi*; cd1, *M. musculus* (CD1); DF, degrees of freedom; myo, *M. brockmani*; SS, sum of squares; swi, *M. musculus* (Swiss Webster).
