# Supplementary material for: Comparative analysis of ear-hole closure identifies epimorphic regeneration as a discrete trait in mammals
Source: Nat Commun. 2016 Apr 25;7:11164. doi: 10.1038/ncomms11164 (PMC4848467; doi:10.1038/ncomms11164)
Supplement: Supplementary Information — Supplementary Figures 1-9 and Supplementary Tables 1-5 [file ncomms11164-s1.pdf]

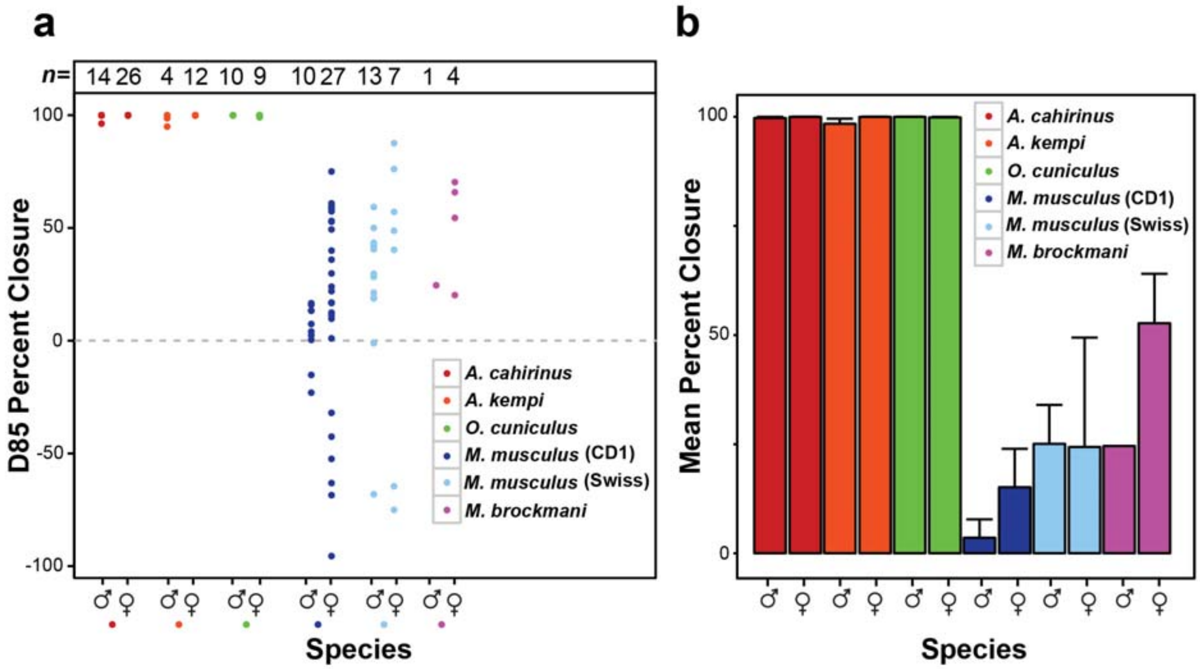

Supplementary Figure 1 | The ability to regenerate an ear hole is discontinuous with wound healing. Ear-hole closure at D85 for each sex within each species observed. Data show a binary response to a 4 mm hole: complete closure (*A. cahirinus*, *A. kempi*, and *O. cuniculus*) or non-closure (*M. musculus* and *M. brockmani*). Data represent individual ear-hole area as a percent of 4 mm diameter hole (a) and mean percent ± SEM of total ear hole closed by D85 (b).

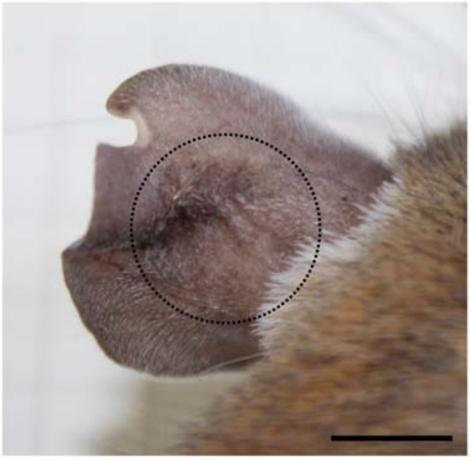

Supplementary Figure 2 | *A. cahirinus* close and regenerate 8 mm ear punches. Representative image showing closure of an 8 mm ear hole 6 months after wounding. Along with complete closure, hair follicles and pigmentation return similar to a 4 mm ear hole. Spiny mice tend to tear a hole this size making it difficult to track closure; however, we have seen closure after 6 months in 6/6 ears that remained intact. Scale bar equals 10 mm and dotted circle indicates initial 8 mm ear hole.

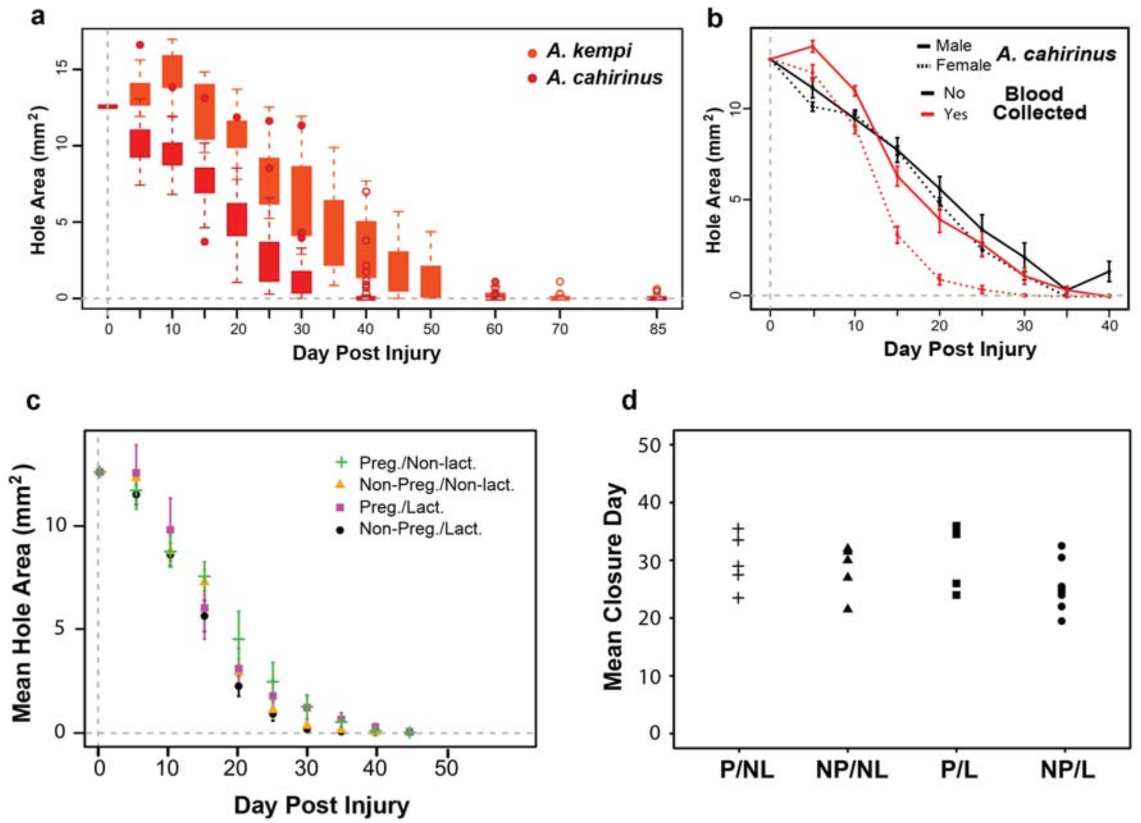

Supplementary Figure 3 | Biotic factors affect regeneration rate in female *A. cahirinus*. (a) Ear closure rates in *A. kempi* and *A. cahirinus* (data includes males and females) showing faster closure in *A. cahirinus*. **(b)** Ear closure rates in male and female *A. cahirinus* with and without blood collected (BC) on D0, 1 and 15. Data represented are box and whiskers (a), and mean and SEM (b). Comparisons showed a significant difference across time between males and females in the BC mice (Repeated measures Two-way ANOVA, F=4.72; H-F-L p-value=0.0058) but no significant difference when blood was not collected (Repeated measures two-way ANOVA, F=2.16; H-F-L p-value=0.0824). There were also significant differences in ear hole closure across time for both males and females when comparing BC/No BC groups (Repeated measures two-way ANOVA, Males: F=12.47, H-F-L p-value<0.0001; Females: F=34.85, H-F-L p-value<0.0001). Ear-hole area over time after a 4 mm ear-hole punch assay in *A. cahirinus* **(c)**. Day of closure for each ear was determined among the experimental groups **(d)**. Data represent individual ears with mean and SEM (c), and mean and SEM (d). We compared four groups of females: (1) pregnant and non-lactating (n = 5), (2) non-pregnant and non-lactating (n = 5), (3) lactating and pregnant (n = 5), and (4) lactating and non-pregnant (n = 8). Comparison of ear-hole closure in these groups over time resulted in no significant interaction of pregnancy, lactation, and day on ear hole area (Repeated Measures Two-way ANOVA; F = 1.23, H-F-L p-value = 0.3055). However, there was a significant effect for lactation across time (Repeated Measures Two-way ANOVA, F=3.08; H-F-L p-value=0.0219), but not for pregnancy (Repeated Measures Two-way ANOVA, F=0.98, H-F-L p-value=0.4221). Lastly, time to complete ear closure was not different among groups (Two-way ANOVA: $F_{3,19} = 1.75$, p = 0.1905; Extended Data Table 3).

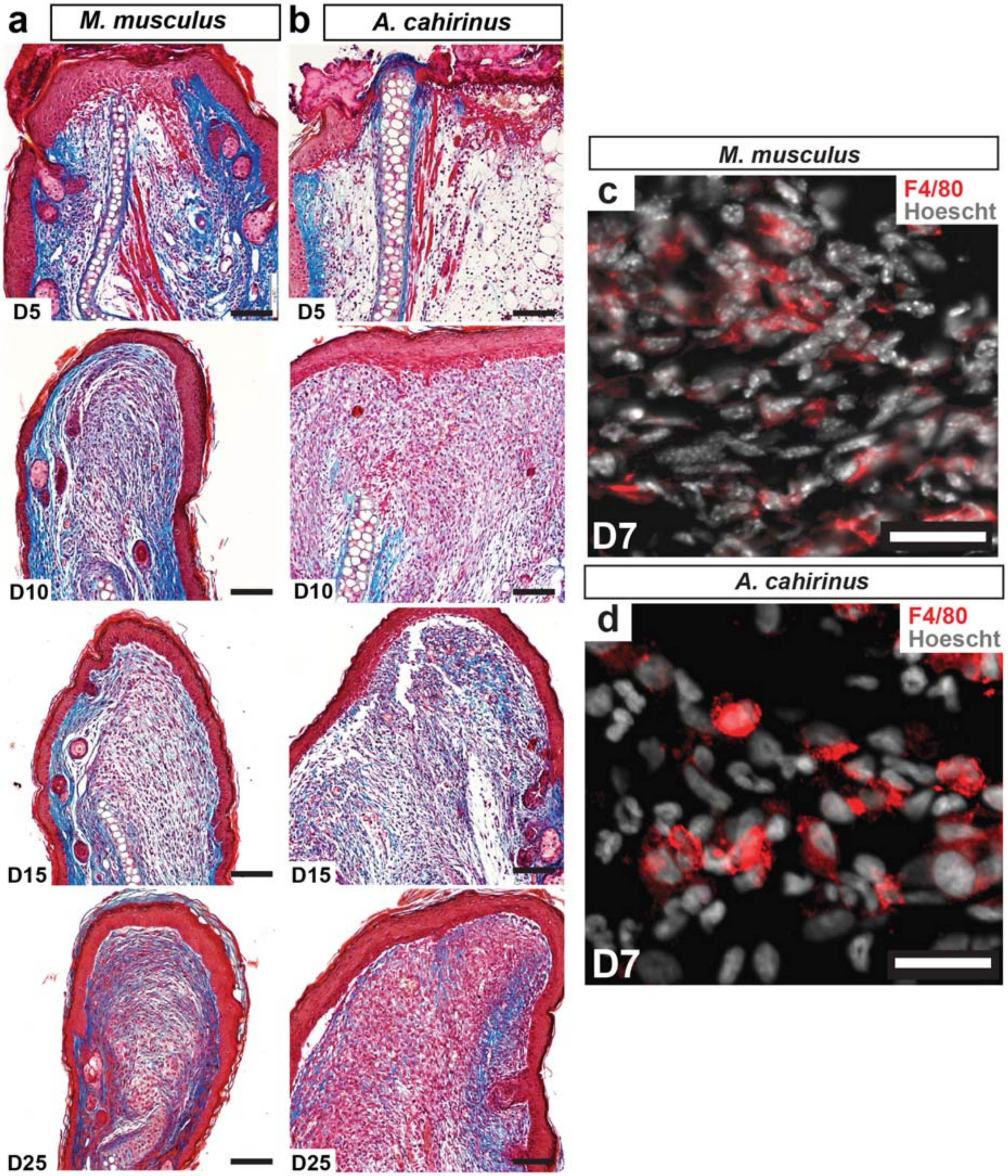

Supplementary Figure 4 | Comparison of healing dermis between M. musculus and A. cahirinus indicates similar early responses to injury and distinct fibroblasts populations associated with regeneration as a later response. (a, b) Histological analysis of healing tissue in *M. musculus* (a) and *A. cahirinus* (b). Mesenchymal cells accumulate to form a blastema in *A. cahirinus* that is distinct from scar tissue in *M. musculus*. Scale bar = 100µm. **(c, d)** Macrophages infiltrate the wound area by D7 in *A. cahirinus* and (c) in *M. musculus* (d). Red = F4/80; Grey = Hoescht.

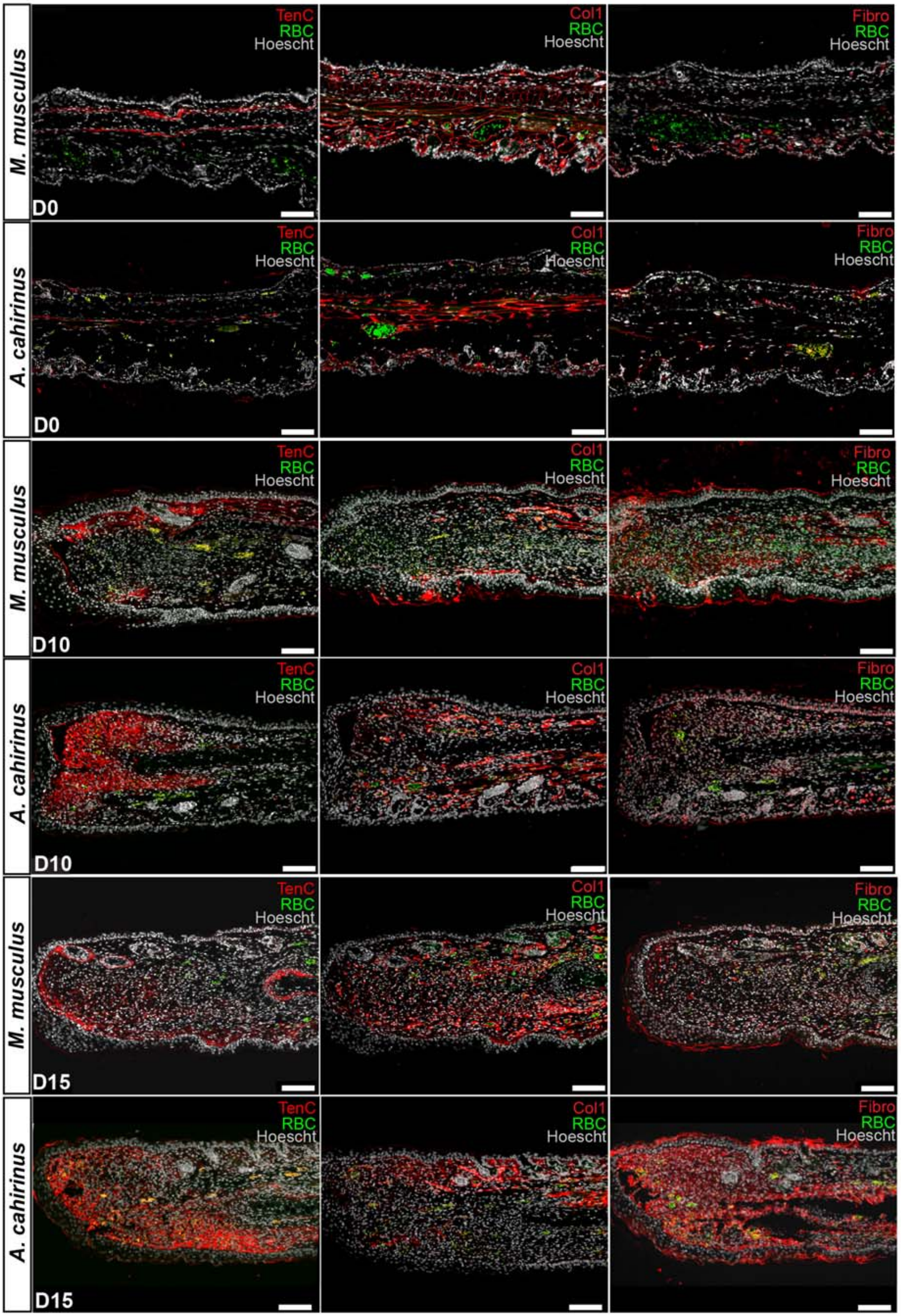

M. musculus
A. cahirinus
M. musculus
A. cahirinus
M. musculus
A. cahirinus
TenC
RBC
Hoescht
Col1
RBC
Hoescht
Fibro
RBC
Hoescht
D0
D0
D10
D10
D15
D15

Supplementary Figure 5 | Immunostaining for the extracellular matrix proteins, Tenascin C, Collagen I and Fibronectin in the total area of protein deposition. Representative images of serial sections for immunohistochemistry to detect Tenascin (TenC), Collagen-I (Col1) and Fibronectin (Fibro) in *A. cahirinus* and *M. musculus* in uninjured tissue (a-f) and at Day 10 after injury (g-l). Nuclei were counterstained with Hoescht (grey), antigen was detected by fluorescent secondary antibody (red), autofluorescent red blood cells (RBC) (green). Scale bar = 100 μm.

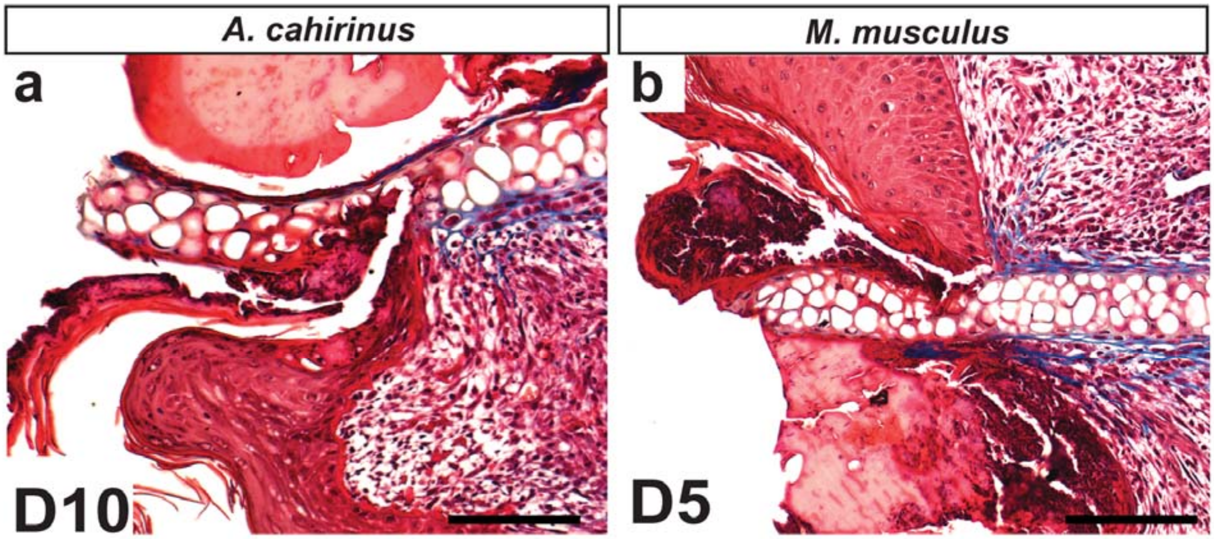

C	Tlme to complete re-epithelialization	
Days Post Injury	*A. cahirinus*	*M. musculus*
5	0/8 (0%)	4/5 (80%)
10	4/8 (50%)	5/5 (100%)
15	4/4 (100%)	3/3 (100%)

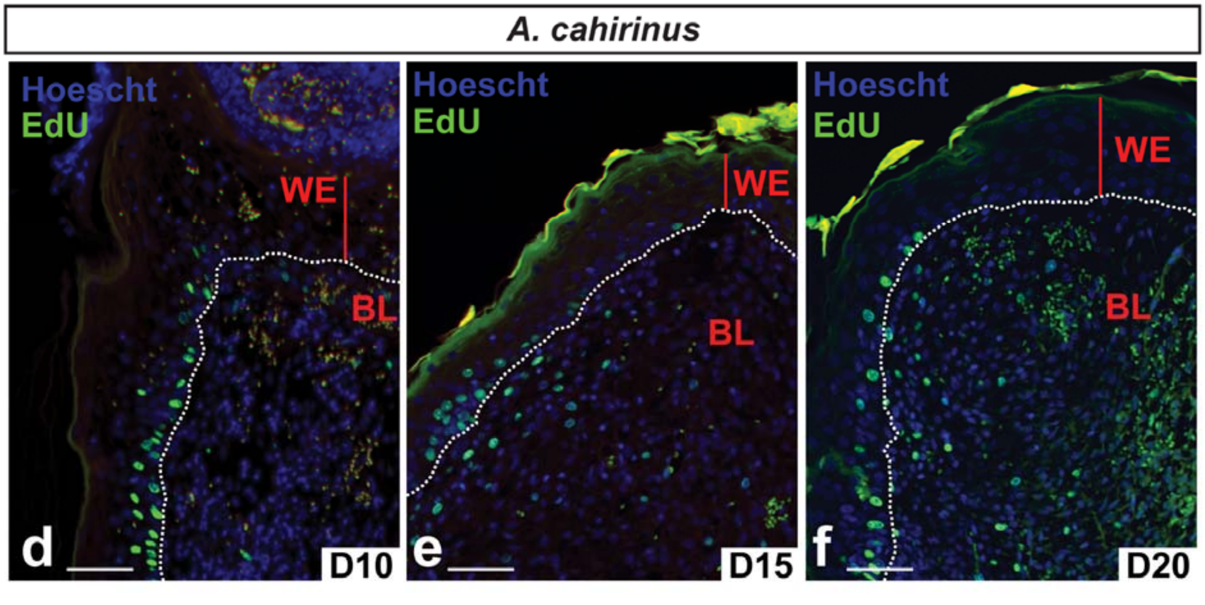

Supplementary Figure 6 | The epidermis between Acomys and Mus is distinctive (a) During the histolysis stage of regeneration, the epidermis cuts a path through the cartilage plate as it migrates to close the wound in *A. cahirinus.* **(b)** Similarly, during the histolysis stage of wound healing in *M. musculus* the epidermis cuts a path through the cartilage plate as it migrates to close the wound. **(c)** Epidermal closure is complete in 80% of Mus by D5 and complete in 100%

of Mus tested by D10. In contrast, 0% of Acomys tested show complete epidermal closure by D5 and only 50% show complete closure by day 10. **(d-f)** EdU+ cells in *A. cahirinus* remain at the epidermal boundary where active cell proliferation generates cells that contribute to the wound epidermis. WE = wound epidermis, BL = blastema. Dotted line marks epidermal/mesenchymal border. Scale bars = 50 µm.

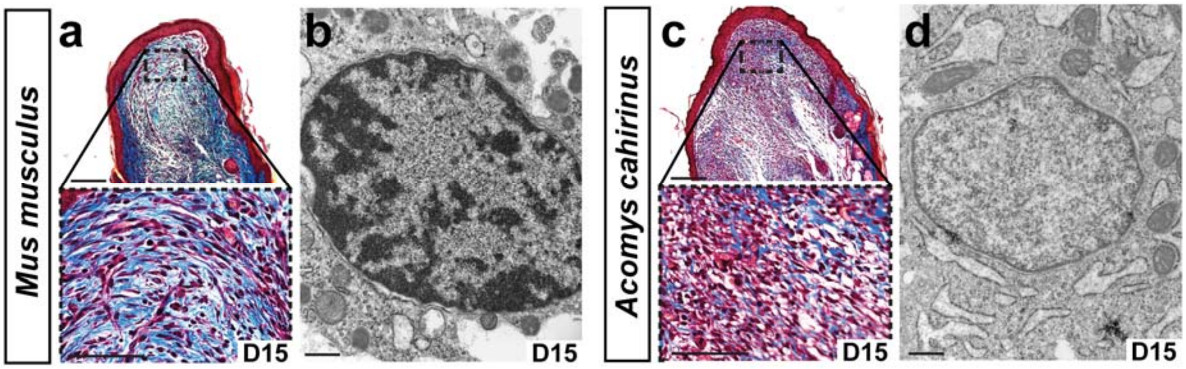

Supplementary Figure 7 | *A. cahirinus* fibroblasts indicate a dedifferentiated morphology and nucleus compared to *M. musculus*. Representative images of healing central tissue showing epidermis and dermis for *M. musculus* (a) and *A. cahirinus* (c)**.** Boxed area is 40x magnification of the dermis at D15. Representative transmission electron microscopy (TEM) images of fibroblasts from D15 dermal area beneath epidermis from *M. musculus* (b) and *A. cahirinus* (d). Scale bars = 100 μm (a, c), 0.6 μm (b, d).

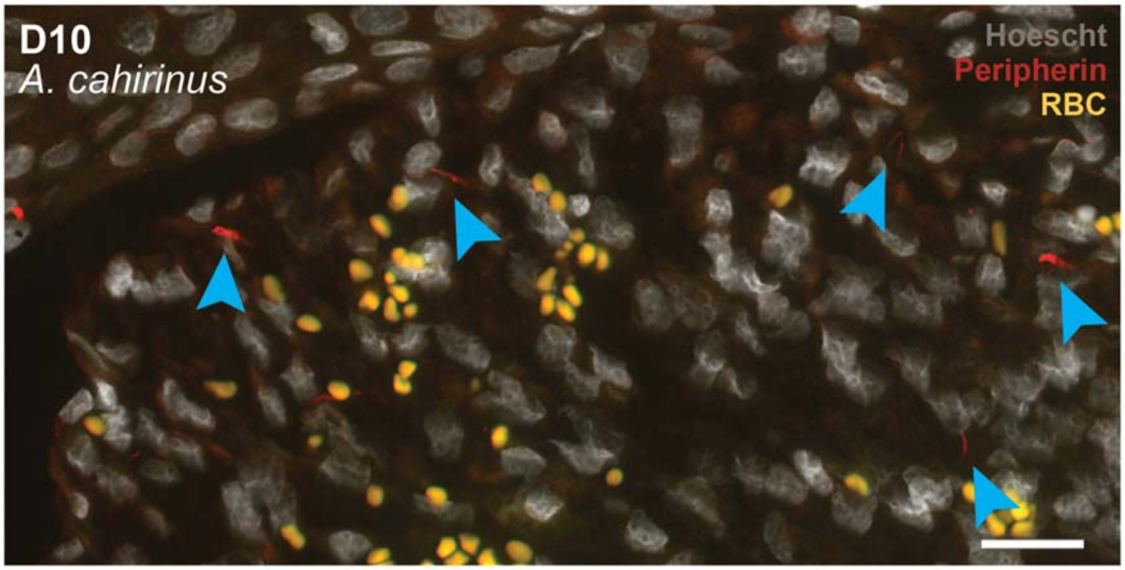

Supplementary Figure 8 | Peripheral nerve axons past amputation plane at D10. Representative image showing presence of axons (arrow heads) into the blastema at D10 in *A. cahirinus*. Image shows IHC for Peripherin (red), autofluorescent red blood cells (yellow) and counter stained with Hoescht to identify nuclei (gray). Scale bar = 20 µm

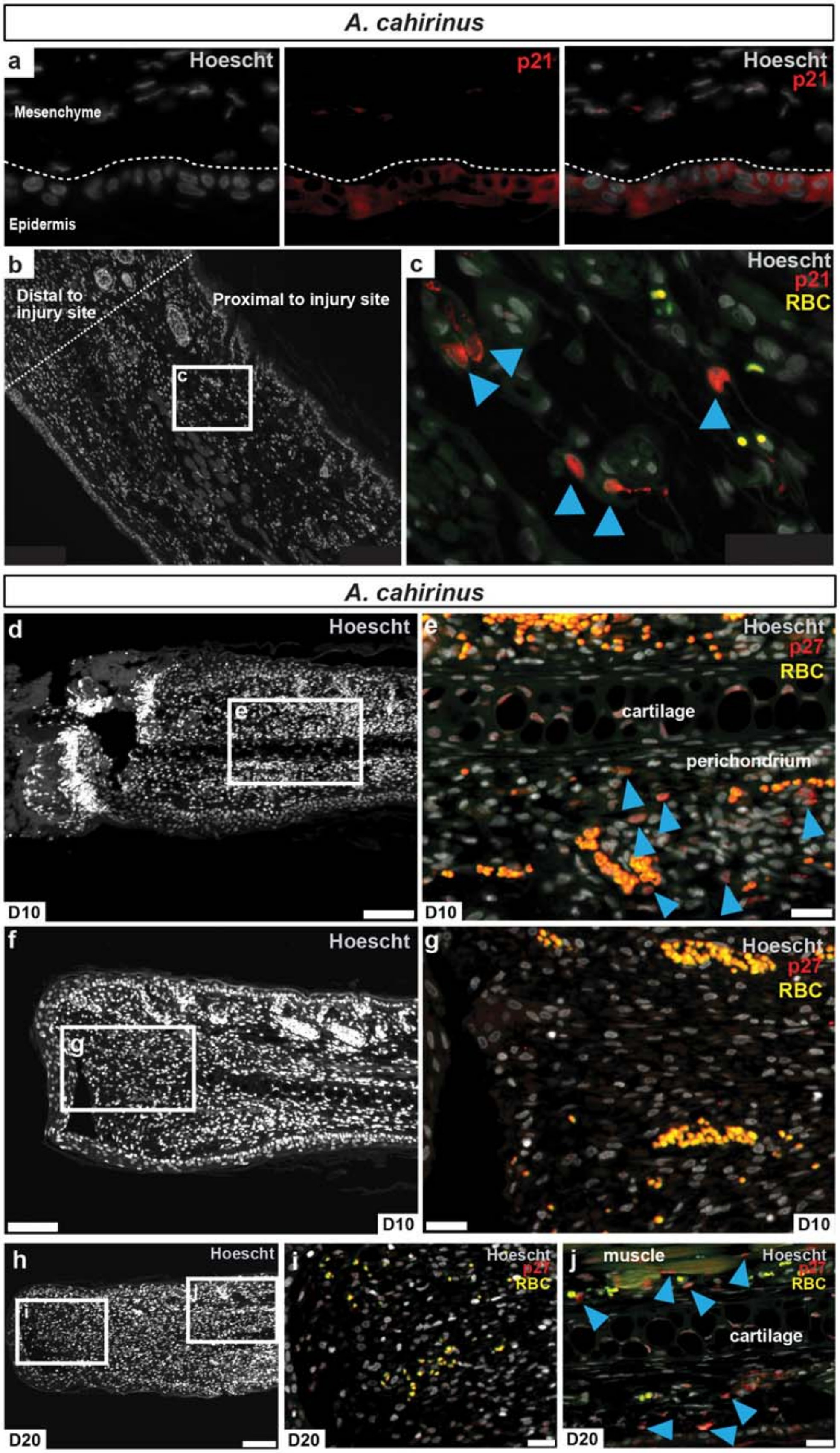

A. cahirinus
a
Hoescht
Mesenchyme
Epidermis
p21
Hoescht
p21
b
Distal to injury site
Proximal to injury site
c
c
Hoescht
p21
RBC
A. cahirinus
d
Hoescht
e
D10
e
Hoescht
p27
RBC
cartilage
perichondrium
D10
f
Hoescht
g
D10
g
Hoescht
p27
RBC
D10
h
Hoescht
i
j
D20
i
Hoescht
p27
RBC
D20
j
muscle
Hoescht
p27
RBC
cartilage
D20

Supplementary Figure 9 | p21 and p27 do not localize to the nucleus of blastema cells in *A. cahirinus* **(a)** Epidermal cells proximal to the injury site display cytoplasmic staining of p21. Red = p21; Grey = Hoescht nuclear stain. **(b)** For orientation, 10x photomicrograph of the area proximal to injury at D25 in *A. cahirinus.* **(c)** Higher magnification view of boxed area in (b) shows nuclear localization of p21 (blue arrows) in mesenchymal cells of the uninjured tissue. Red = p21; Grey = Hoescht; Yellow = autofluorescing red blood cells (RBC). Ratio of gene expression derived from RNA-seq data comparing *collagen1a1* versus *collagen3a1* between *M. musculus* (CD1) (blue) and *A. cahirinus* (red). **(d)** Low magnification of *A. cahirinus* ear at D10 showing the orientation of the wound site (left) and proximal uninjured cartilage (box). **(e)** High magnification of the boxed area in (a). Cells of the cartilage, perichondrium, and dermis show positive nuclear staining of p27 (arrows). **(f)** Low magnification of the ear at day 10 showing the orientation of the injury area (box). **(g)** High magnification of boxed area in (e). Cells at the injury site are negative for p27. **(h)** Low magnification of the ear at D20 showing the orientation of the injury site **(i)** and proximal uninjured tissue **(j).** (g) High magnification of boxed area in (i). Cells at the injury site are negative for p27 while cells in the same section proximal to the injury site (j) are positive for p27. Cells of the uninjured muscle, cartilage, and dermis show positive nuclear staining. RBC = red blood cells. Scale bars in d, f, h = 100μm and in e, g, i, j = 20μm.

Supplementary Table 1 | Four-millimeter punch assay groups.

Group	Species	Location	Obtained	Blood Collected?	Females	Males	Total
CD-1	*Mus musculus*	Kentucky	Harlan	No	27	10	37
Swiss Webster	*M. musculus*	Kenya	Local breeder	Yes	7	13	20
Myomyscus	*Myomyscus brockmani*	Kenya	Live trap	Yes	4	1	5
Cahirinus	*Acomys cahirinus*	Kentucky	Colony	No	26	14	40
Kempi	*A. kempi*	Kenya	Live trap	Yes	12	4	16

Other groups (not included in model)

New Zealand White Rabbit	*Oryctolagus cuniculus*	Kenya	Local breeder	No	9	10	19
MRL/MpJ	*M. musculus*	Florida	Gift from Dr. Edward Scott	No	3	7	10
BC Cahirinus	*A. cahirinus*	Kentucky	Colony	Yes	10	10	20

Supplementary Table 2 | Testing for sex effects within species (Repeated measures Two-way ANOVA).

Species	Contrast	DF	Contrast SS	Mean Square	F-Value	Adjusted p-value (H-F-L)
A. cahirinus	Day*sex	5	14.0773	2.8155	2.16	0.0824
A. kempi	Day*sex	5	80.9725	16.1945	8.73	0.0003
CD1	Day*sex	5	33.2563	6.6513	2.44	0.0692
Swiss Webster	Day*sex	5	24.1677	4.8335	1.12	0.3457
M. brockmani	Day*sex	5	15.5758	3.1152	1.23	0.3516

Supplementary Table 3 | Lactation but not pregnancy affects regeneration rate.

Group	Description	n (mice)	Day completely closed (median)	Day completely closed (mean ± SEM)
A	Pregnant, not lactating	5	29.5	29.8 ± 1.90
B	Not pregnant, not lactating	5	27.0	28.4 ± 1.90
C	Pregnant, lactating	5	29.5	31.1 ± 1.90
D	Not pregnant, lactating	8	25.5	25.4 ± 1.50

Repeated Measures ANOVA Results:

	DF	Type III SS	Mean Square	F Value	H-F-L p-value
**Day**	5	2196.8029	439.3606	442.31	<.0001
**Day*pregnant**	5	4.8698	0.9740	0.98	0.4221
**Day*lactate**	5	15.2983	3.0597	3.08	0.0219
**Day*pregnant*lactate**	5	6.1100	1.2220	1.23	0.3055
**Error(Day)**	95	94.3661	0.9933		

Supplementary Table 4 | qPCR Primers

Gene	NCBI Ascension (*Mus*)<br>Trinity Contig (*Acomys*)	Forward (5’ -> 3’)<br>Reverse (5’ -> 3’)	PCR Product Size (bp)
Mus musculus			
Tbp	NM_013684.3	CCCTGGTCCCTCTGGAAATG<br>GGACCTTTGCCCCCTTCTTT	88
Col1a1	NM_007742.4	CACGGACAACTGCGTTGATTT<br>TGTTCTTCACTCTTGGCTCCTG	51
Col3a1	NM_009930.2	CTCGAGGCAATGATGGTGCT<br>TCCTGGTGAGCCATTTGAGC	178
Tnc	NM_011607.3	GAGCCAGGGCAAGAATACACTG<br>AGATTTTCCAGGGAAGGCACTTCTT	110
Fn1	NM_010233.2	GGAGCCTTCACACATCACCA<br>GTGGCCAGGAATGGTAGCTT	88
Mmp9	NM_013599.3	CGACATAGACGGCATCCAGTA<br>ACATAGTGGGAGGTGCTGTC	107
Mmp13	NM_008607.2	TGATGGGATTCCCTGGACCT<br>CCAGCCTTTCCAGGTTCTCC	52
Acomys cahirinus			
Tbp	comp453643_c1_seq8	CTGCGCTGATTTTCAGTTCTGG<br>AGCTTCTGCACAACCCGA	103
Col1a1	comp465062_c1_seq198	TGGACCCAAGGGTACTGCT<br>GAACACCACGCTCTCCAGAC	53
Col3a1	comp465069_c3_seq100	TGGCATTCCTGGATTCCCTG<br>GAGCACCTGGTTCACCCTTT	80
Tnc	comp464237_c9_seq84	GAGAAGGGCAGGCACAAGAG<br>TCAGATTTTCCAGGGACGGC	76
Fn1	comp465100_c4_seq33	AACGCACCGGAACCATCAC<br>AGGAGTTAAGGTGGCCTGGAA	106
Mmp9	comp450929_c2_seq2	TGGTCATGCACTGGGCTTAG<br>CTTGGGTCAGGCTTAGGGC	150
Mmp13	comp455856_c3_seq8	GCCATTACCAGTCTCCGAGG<br>ACACGGTTGGGAAGTTCTGG	128

Supplementary Table 5 | Antibodies

Antigen	Animal	Company	Catalog number
Keratin 17	Rabbit anti mouse	Abcam	ab53707
pHH3	Rabbit anti mouse	Cell Signaling	9701
pRb	Rabbit anti mouse	Cell Signaling	8516
p21	Rat anti mouse	Centro Nacional de Investigaciones Oncologicas	HUGO291
p27	Rat anti mouse	Centro Nacional de Investigaciones Oncologicas	SON82B
Ki67	Rabbit anti mouse	Abcam	ab15580
Peripherin	Rabbit anti mouse	Millipore	AB1530
Tenascin C	Rabbit anti chicken	Abcam	ab19013
Fibronectin	Rabbit anti mouse	Abcam	ab23750
Collagen 1	Rabbit anti mouse	Abcam	ab34710
F4/80	Rat anti mouse	eBiosciences	14-4801
EdU	Click-IT EdU imaging kit	Invitrogen	A10044
