# Supplementary material for: Comparative analysis of ear-hole closure identifies epimorphic regeneration as a discrete trait in mammals
Source: Nat Commun. 2016 Apr 25;7:11164. doi: 10.1038/ncomms11164 (PMC4848467; doi:10.1038/ncomms11164)
Supplement: Peer Review file [file ncomms11164-s3.pdf]

Editorial Note: this manuscript has been previously reviewed at another journal that is not operating a transparent peer review scheme
